# A natural DYRK1A inhibitor as a potential stimulator for β‐cell proliferation in diabetes

**DOI:** 10.1002/ctm2.494

**Published:** 2021-07-19

**Authors:** Mengzhu Zheng, Qingzhe Zhang, Chengliang Zhang, Canrong Wu, Kaiyin Yang, Zhuorui Song, Qiqi Wang, Chen Li, Yirong Zhou, Jiachun Chen, Hua Li, Lixia Chen

**Affiliations:** ^1^ Hubei Key Laboratory of Natural Medicinal Chemistry and Resource Evaluation, School of Pharmacy, Tongji‐Rongcheng Center for Biomedicine, Tongji Medical College Huazhong University of Science and Technology Wuhan China; ^2^ Wuya College of Innovation, Key Laboratory of Structure‐Based Drug Design & Discovery, Ministry of Education Shenyang Pharmaceutical University Shenyang China; ^3^ Department of Pharmacy, Tongji Hospital, Tongji Medical College Huazhong University of Science and Technology Wuhan China

Dear Editor,

Dual‐specificity tyrosine‐phosphorylation‐regulated kinase 1A (DYRK1A) was demonstrated as a promising therapeutic target for diabetes for its influence on pancreatic β‐cell mass and proliferation.^1^, ^2^, ^3^, ^4^, ^5^, ^6^, ^7^, ^8^ In this study, we identified desmethylbellidifolin (DMB) as a novel and potent DYRK1A inhibitor. It was found to stimulate proliferations of β‐cell both in vitro and in vivo via targeting DYRK1A.

Twelve xanthone compounds (1‐12; Figure S1A) from *Swertia* species were selected by molecular docking for further testing (Table S1). Among them, DMB has the lowest *K*_d_ and *IC*_50_ values. The equilibrium dissociation constant (*K*_d_) of DMB (compound 1, 5.11 ± 0.33 μM) with DYRK1A (Figure 1A) was much smaller than that of harmine (81.7 ± 12.8 μM) (Figure 1B,C), suggesting a strong binding (Table S1, Figure S1B). Enzymatic activities assay further confirmed that DMB strongly inhibited DYRK1A, with lower *IC*_50_ values than harmine (Table S1).

**FIGURE 1:**
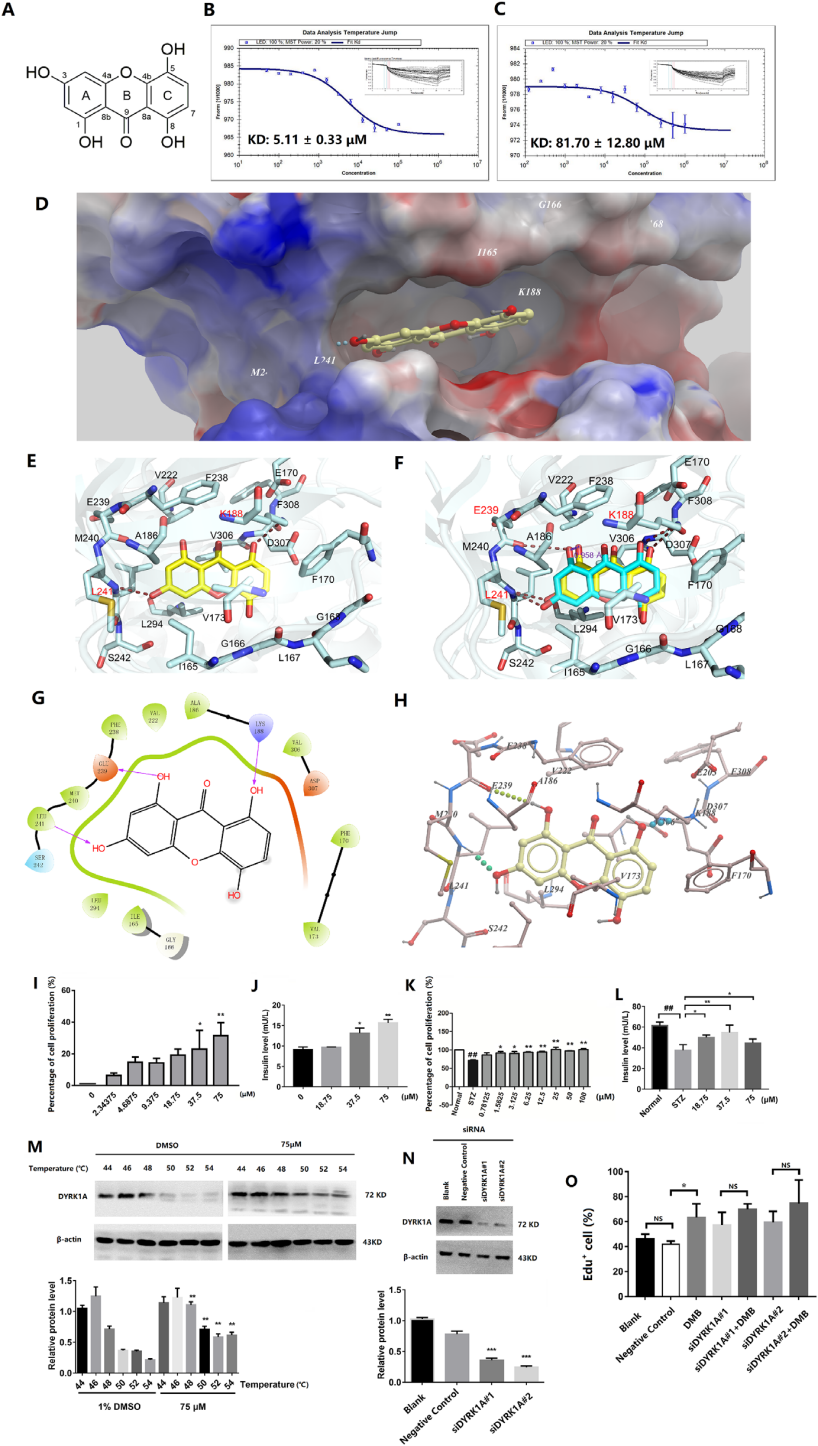
DMB was bound to DYRK1A directly and promoted INS‐1 cell proliferation and regeneration. (A) Molecular structure of DMB with atoms numbered. (B, C) Measurement of binding affinity of DMB and harmine to DYRK1A by MST. The binding curves were shown. (D) The overall view of DMB in the binding pocket. The protein was shown as the potential surface. (E) Close‐up view of the DMB binding pocket. DMB was shown as yellow sticks; the residues involved in inhibitor binding are shown as light blue sticks. Hydrogen bonds with Lys188 and Leu241 were shown as orange dashed line. (F) Comparison of the binding modes between docking model and the crystal structure for DMB‐DYRK1A complex. DMB in the docking model was shown as cyan sticks; DMB in the crystal structure was shown as yellow sticks. Hydrogen bonds with Lys188, Glu239, and Leu241 were shown as orange dashed line. (G) Ligand interaction diagram of DMB with DYRK1A. (H) Low‐energy binding conformations of DMB bound to DYRK1A generated by virtual ligand docking. (I) The proliferation percentage of INS‐1 cells in different DMB treated groups. The proliferation percentages were about 22.94% and 31.46% at 37.5 and 75 μM of DMB, respectively, which were higher than that of control. (J) The insulin level in the supernatant of the culture medium was significantly increased after treatment of DMB with the concentration of 18.75‐75 μM. (K) DMB could promote the proliferation of β‐cell in the concentration range of 0‐100 μM in STZ‐induced cell damage model. (L) The insulin level in the supernatant of the culture medium was significantly increased after treatment of DMB with the concentration of 18.75‐75 μM in STZ‐induced cell damage model. (M) Up: CETSA was performed on INS‐1 cells. The stabilizing effects of DMB on DYRK1A and β‐actin at different temperatures were evaluated by Western blot analysis. Below: Analysis of Western blot results in (M). (N) Up: Knockdown of DYRK1A in INS‐1 cells was established. The expression of DYRK1A was determined by Western blot. Below: Analysis of Western blot results in (N). (O) DAPI and EdU double staining was used to determine the proliferation of INS‐1 cells in different treated groups. There was no significant difference between siDYRK1A^#^1/^#^2 and siDYRK1A^#^1/^#^2 + DMB groups. All data are the average of three experiments (*n* = 3). The results are presented as the mean ± SD; NS: no significant difference, *P < .05, **P < .01, by one‐way analysis of variance (ANOVA). Experiments were performed in triplicates

The crystal structure of DMB‐DYRK1A complex was solved at 2.7 Å (PDB ID 6LN1, Table S2). DMB was bound with DYRK1A at the same binding pocket as harmine (Figures 1D, S1C), adopted an extended conformation and occupied the whole flat‐shaped pocket (Figures 1D, S1D). Hydrogen bonds were formed between 3‐hydroxyl and Leu241, also 8‐hydroxyl and Lys188. Besides, there were hydrophobic interactions between DMB with Phe170, Leu294, Val306, and Phe238 (Figure 1E,F). Interestingly, the conformation of DMB in crystal structure was almost completely overlapping with that predicted by docking (Figure 1F). In crystal structure, DMB rotated approximately 10 degree toward the direction of Phe170 with 1‐hydroxyl oxygen shifted 0.958 Å, impairing docking predicted hydrogen bonding with Glu239 (Figure 1G,H). However, the shorter bond lengths of other two hydrogen bonds, and the closer distance to the hydrophobic environment of Phe170, favored the real conformation of DMB in the crystal structure (Figure 1F, Table S3).

DMB stimulated proliferation of INS‐1 cell with function and capability of insulin secretion (Figure 1I,J). The number of EdU^+^ INS‐1 cells in treated group was markedly increased (Figures S2A,B, S3). Even for INS‐1 cells treated by STZ, the number of proliferating cells was increased after co‐incubation with DMB for 24 hours (Figures 1K,L, S2C,D, S3).

DMB (75 μM) increased the thermal stability of DYRK1A in intact cells (Figure 1M). Administration of DMB in siDYRK1A ^#^1 and siDYRK1A ^#^2 groups did not further enhance proliferations of INS‐1 cells (Figures 1N,O, S3), suggesting that DMB‐induced proliferations of INS‐1 cells depended on targeting DYRK1A.

Compared to cytoplasmic NFATc1 in INS‐1 cells, DMB treatment induced increase in nuclear NFATc1, which indicated that DMB may stimulate translocation of NFATc1 from the cytoplasm to the nucleus (Figure 2A). NFATc proteins were translocated into the nucleus to alter gene expression, ultimately activating transcription of insulin‐related genes such as *Ccnd1*, *Ccnd2*, *Ccnd3*, and *CDK4*.^9^ RT‐PCR revealed that levels of *Ccnd1*, *Ccnd2*, and *Ccnd3* mRNA were dose‐dependently upregulated after DMB treatment (Figure 2B). By treatment with DMB, expression levels of cell‐cycle inhibitors (eg, p15^INK4^, p16^INK4^, and p57^CIP2^) were lower, whereas expression levels of relevant cyclins and CDKs (eg, cyclin A, CDK1) remained unchanged (Figure 2C). E2F1 expression was increased but not in dose‐dependence, and immunofluorescence results further confirmed the increase of E2F1, suggesting that DMB could promote cell cycles of INS‐1 cells (Figure 2D).

**FIGURE 2:**
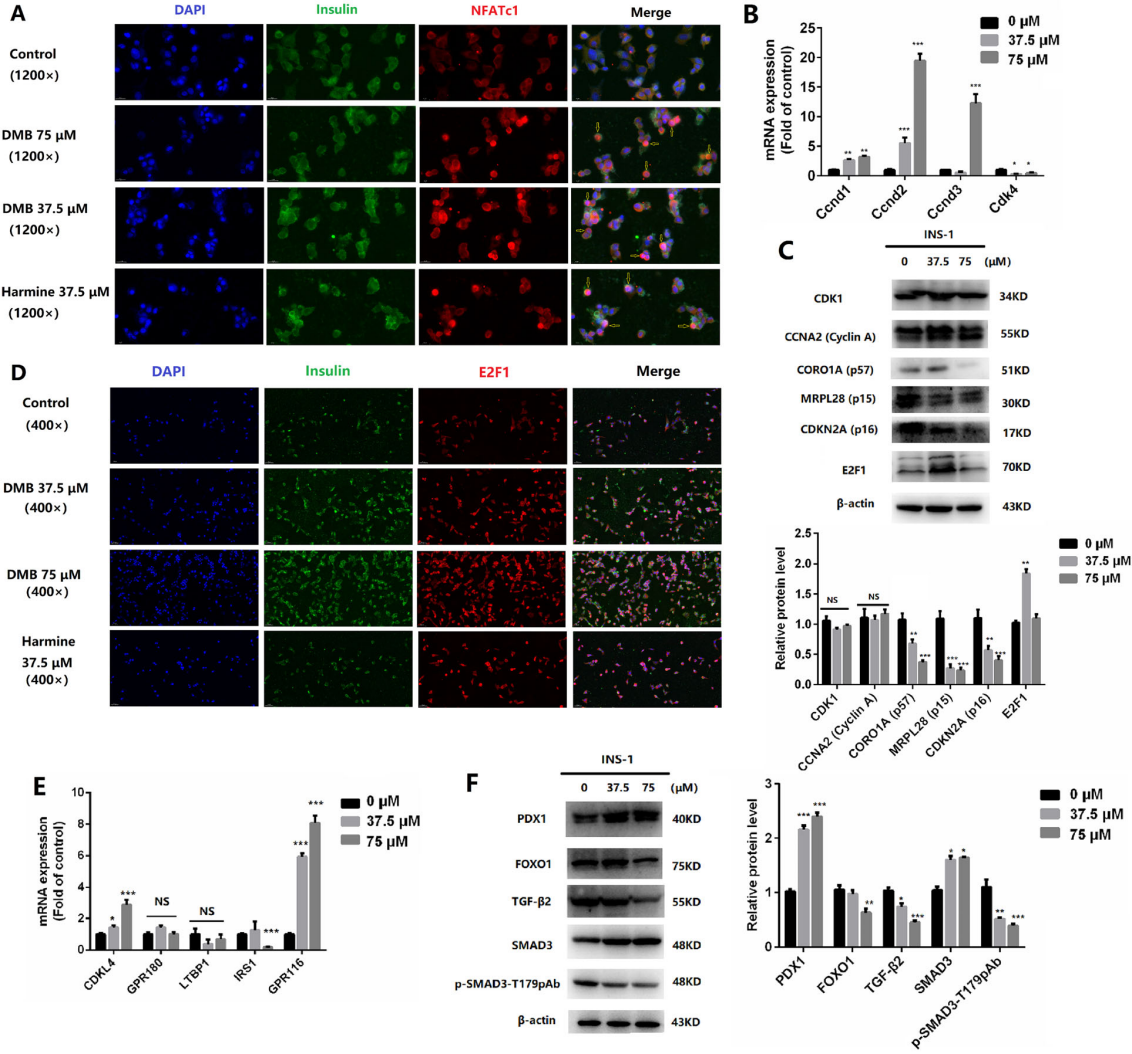
DMB regulated NFATc1 nuclear localization and stimulated INS‐1 cell proliferation involved in calcineurin/NFAT/DYRK1A signaling pathway. (A) Representative images of the effects of DMB (37.5 and 75 μM) and harmine (37.5 μM) treatment on NFATc1 nuclear localization of INS‐1 cells. (Magnification 1200×, the scale bar indicates 20 μm.) (B) The percentage of mRNA expression level of proliferation‐related factors (*Ccnd1*, *Ccnd2*, *Ccnd3*, and *CDK4*) after DMB administration compared with control (*n* = 3, ^*^ *P* < .05, ^**^ *P* < .01, ***P < .001). (C) Representative Western blot of key cell cycle molecules in INS‐1 cells treated with DMB. Protein levels were quantified using grey value analyses by Image J software in the right. (*n* = 3, *P < .05, **P < .01, ***P < .001) (D) Representative images of immunofluorescence in INS‐1 cells for E2F1 in response to DMB treatment (Magnification 400×, the scale bar indicates 50 μm.) (E) Relative gene expression levels determined by qPCR after treatment with DMSO or DMB for 72 hours (*n* = 3, *P < .05, **P < .01, ***P < .001). (F) Western blot analysis of PDK1, FOXO1, TGF‐β2, SMAD3, and phospho‐SMAD3 after treatment with DMSO or DMB for 72 hours. Protein levels were quantified using grey value analyses by Image J software in the right (*n* = 3, *P < .05, **P < .01, ***P < .001)

Gene expression profiling revealed that DMB strongly up‐regulated *CDKL4*, *GPR116*, and *INHBE*, and downregulated *GPR180*, *IRS1*, *DCN*, *LTBP1*, and *SP1* (Table S4). These genes are involved in β‐cell proliferation and blood glucose regulation. Gene expression levels of *CDKL4*, *IRS1*, and *GPR116* were confirmed by RT‐PCR (Figures S4A, 2E). The KEGG revealed that TGF‐β signaling pathway was involved in β‐cell proliferation (Figure S4B). Previous research also revealed combined inhibition of DYRK1A and TGF‐β signaling generates further synergistic increases in β‐cell proliferation.^10^ DMB treatment led to a reduction in SMAD3 phosphorylation and a concomitant increase in SMAD3 abundance. Furthermore, PDX1 expression in INS‐1 cells was increased, while FOXO1 expression was reduced after DMB treatment (Figure 2F). These gene expression changes induced by DMB were consistent with cellular proliferation.

DMB‐induced inhibition of DYRK1A activated proliferation of β‐cells that yielded double‐labeled Ki67^+^ and insulin^+^ nuclei (Figure S5A). DMB also induced p‐histone‐H3, a marker of cell‐cycle transition. The proliferation‐related indicators, p‐P38 and p‐erk, were increased at the protein levels in β‐cells (Figure S5B). β‐cell death or DNA damage induced by DMB was not observed by p‐γ‐H2AX labeling (Figure S6).

The β‐cell mass and size were higher in DMB‐ and harmine‐treated db/db mice groups, insulin/EdU double‐positive cells were a little higher in DMB‐treated group (Figure S7A,B). The number of insulin/Ki67 double‐positive cells was approximately four‐fold higher increased after DMB treatment (Figure S7C,D).

For groups of db/db mice treated with DMB, harmine, and metformin, the 6‐hour fasting blood glucose levels and symptoms of diabetes were ameliorated (Figures 3A, S8, S9), with no significant changes of body weight (Figures 3B). The glucagon and insulin were gradually restored to normal levels after DMB treatment (Figure 3C).

**FIGURE 3:**
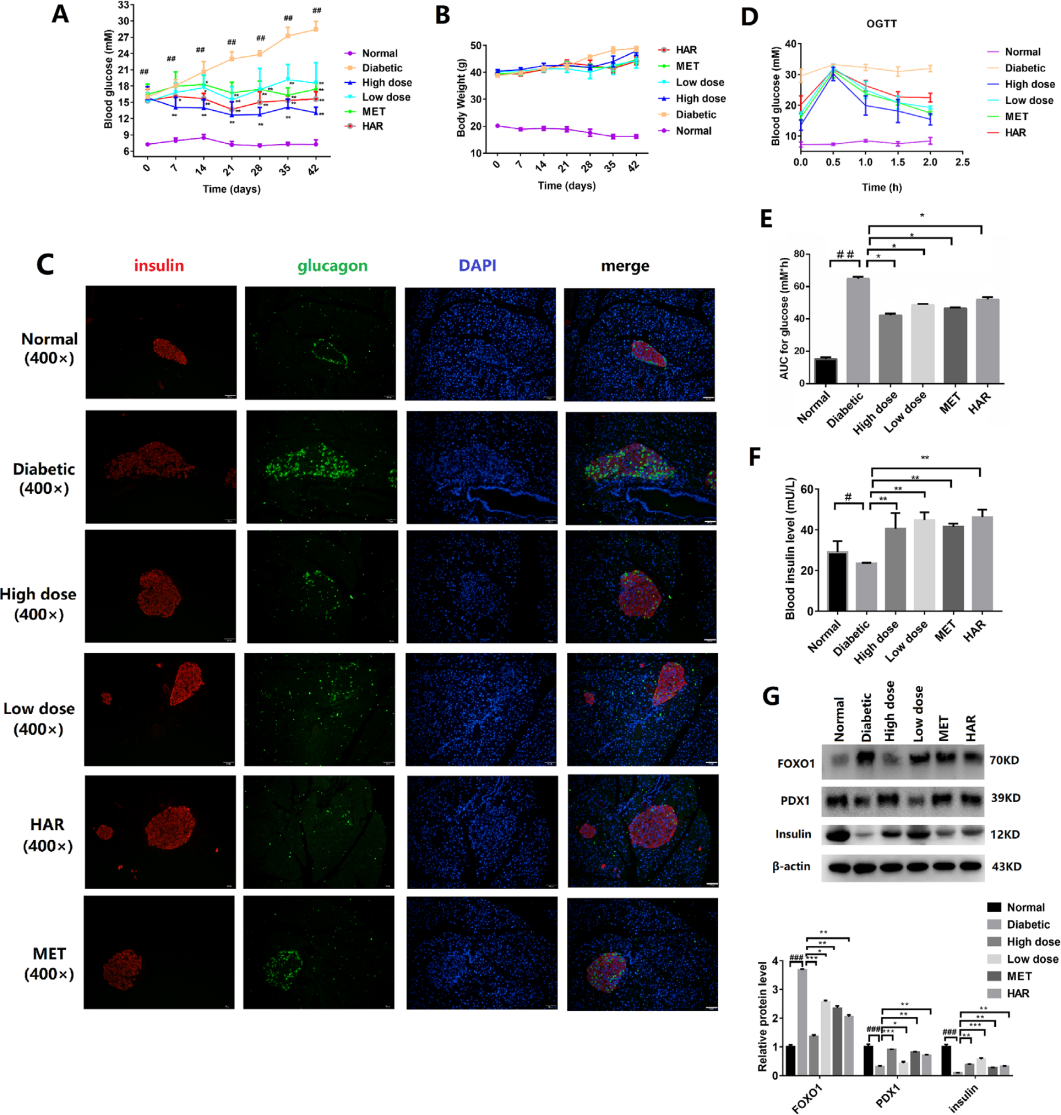
Effects of DMB on markers of β‐cells proliferation and glycemic control in db/db mice in vivo. (A) Blood glucose concentrations measured weekly for 6 weeks. (B) Body weight increased steadily in each administration group. There was no significant difference. (C) Double‐immunofluorescence analysis of the expression level of insulin (green) and glucagon (red) in pancreas tissue (Magnification 400×, the scale bar indicates 50 μm). (D) The blood glucose level of OGTT (mM). (E) The AUC of OGTT. (F) Quantification of blood insulin level. Data are presented as mean ± SD (*n* = 8). ^#^ *P* < .05, ^##^ *P* < .01, compared to the normal group; ^*^ *P* < .05, ^**^ *P *< .01, compared to the diabetic group. Metformin (200 mg/kg) and harmine (200 mg/kg) were used as positive controls. DMB low‐dose group: 75 mg/kg; DMB high‐dose group: 150 mg/kg. (G) The expression level of FOXO1, PDX1, and insulin in pancreatic tissue by Western blot. DMB induced production of PDX1 and decreased FOXO1 expression. Protein levels were quantified using grey value analyses by Image J software in the right (*n* = 3, *P < .05, **P < .01, ***P < .001)

The blood glucose levels in all treated mice was decreased from 30 minutes after drug administration, and the areas under the curves (AUCs) of the DMB‐treated groups were reduced (Figure 3D,E). The serum insulin levels of treated group were increased at the sixth week after drug administration (Figure 3F). Furthermore, DMB partially ameliorated dyslipidaemia and antioxidative stress in db/db mice (Figure S10, Table S5). After treated with DMB, expressions of increased PDX1 and insulin, and reduced FOXO1 were consistent with the enhanced antioxidative capacity in serum (Figure 3G).

In conclusion, DMB from *Swertia bimaculata* was found to be a novel and potent DYRK1A inhibitor. The crystal structure of DYRK1A‐DMB complex revealed that hydrogen bonding and hydrophobic interactions enabled the shape‐driven binding mode of DMB onto DYRK1A. Finally, we elucidated additional β‐cell replication pathways, including Ca^2+^/CaN/NFAT/DYRK1A and TGF‐β signaling pathways, which were mediated by DMB‐dependent DYRK1A inhibition. Compared to these known DYRK1A inhibitors, DMB exhibits better druggability, with low toxicity and broad availability. Next, DMB as a stimulator for β‐cell proliferation will be combined with other classes of drugs to achieve therapeutic effect on diabetes.

## AUTHOR CONTRIBUTIONS

LXC, HL and JCC designed the research and wrote the manuscript. MZZ, QZZ, and CLZ performed the research and acquired the data. CRW, KYY, ZRS, QQW and CL participated the experiments. All authors made substantial contributions to the analysis and interpretation of data. All authors were involved in drafting the manuscript and all approved the final version. HL is responsible for the integrity of the work as a whole.

## FUNDING INFORMATION

This work was supported by the National Natural Science Foundation of China (NSFC) (No. 81773594, 81773869, 81773637, and U1803122), the Fundamental Research Fund for the Central Universities (grant number 2017KFYXJJ151), and Liaoning Revitalization Talents Program (No. XLYC1807182), Wuhan COVID‐19 Rapid Response Call Fund (No. EX20C02), Chunhui Program‐Cooperative Research Project of the Ministry of Education, Liaoning Province Natural Science Foundation (2020‐MZLH‐31), and China Postdoctoral Science Foundation (grant number 2020T130039ZX).

## CONSENT FOR PUBLICATION

All the authors consent for publication.

## CONFLICT OF INTEREST

The authors declare no conflict of interest.

## DATA AVAILABILITY STATEMENT

The datasets used or analyzed in this study are available from the corresponding author on reasonable request.

## Supporting information

Figure S1

Figure S2

Figure S3

Figure S4

Figure S5

Figure S6

Figure S7

Figure S8

Figure S9

Figure S10

Supporting Information 1

Supporting Information 2

Supporting Information 3
