# Supplementary figures and images for: A natural DYRK1A inhibitor as a potential stimulator for β‐cell proliferation in diabetes
Source: Clin Transl Med. 2021 Jul 19;11(7):e494. doi: 10.1002/ctm2.494 (PMC8288015; doi:10.1002/ctm2.494)

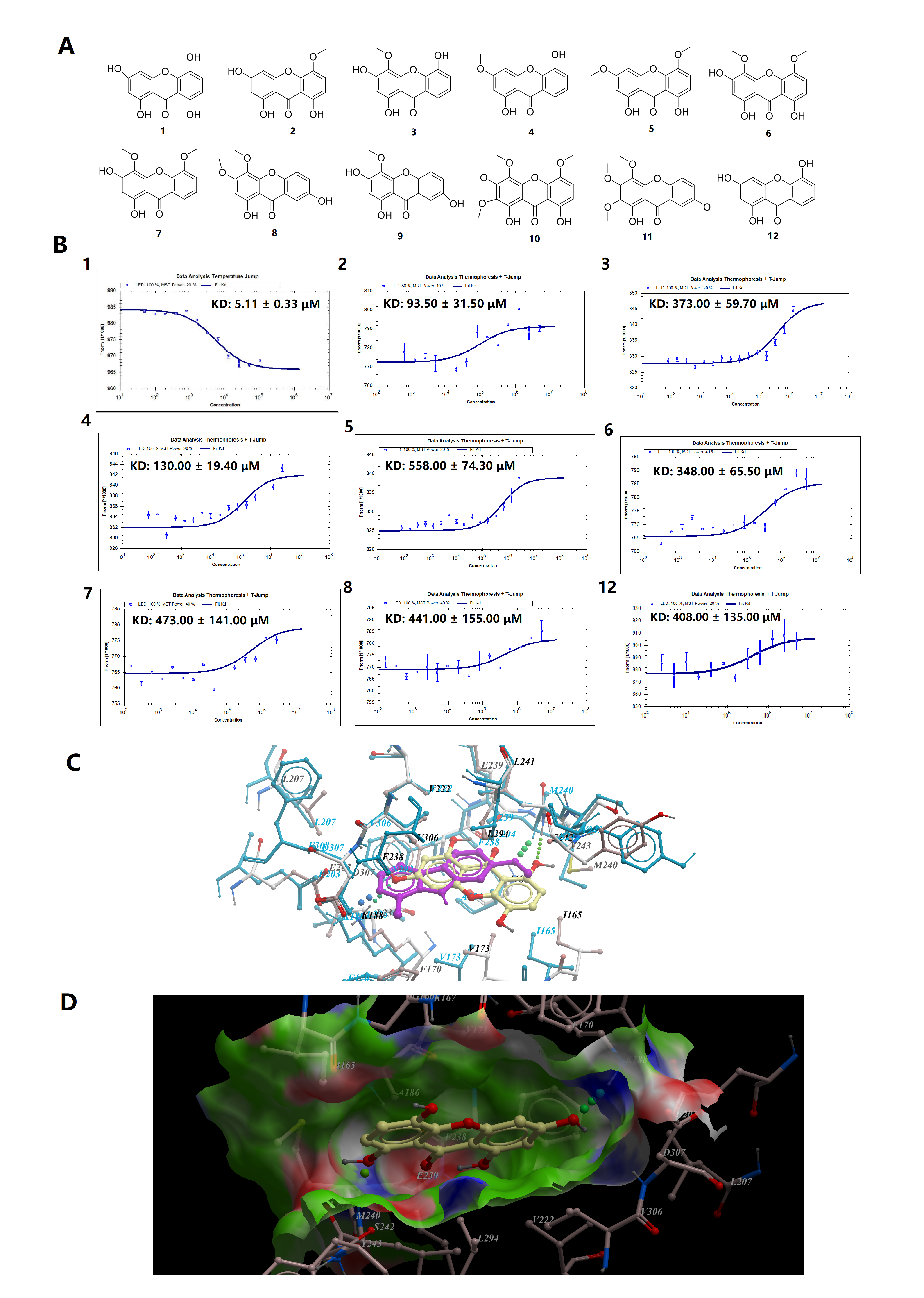

Supplement: Supplementary file 1 — Figure S1 [file CTM2-11-e494-s012.tif]

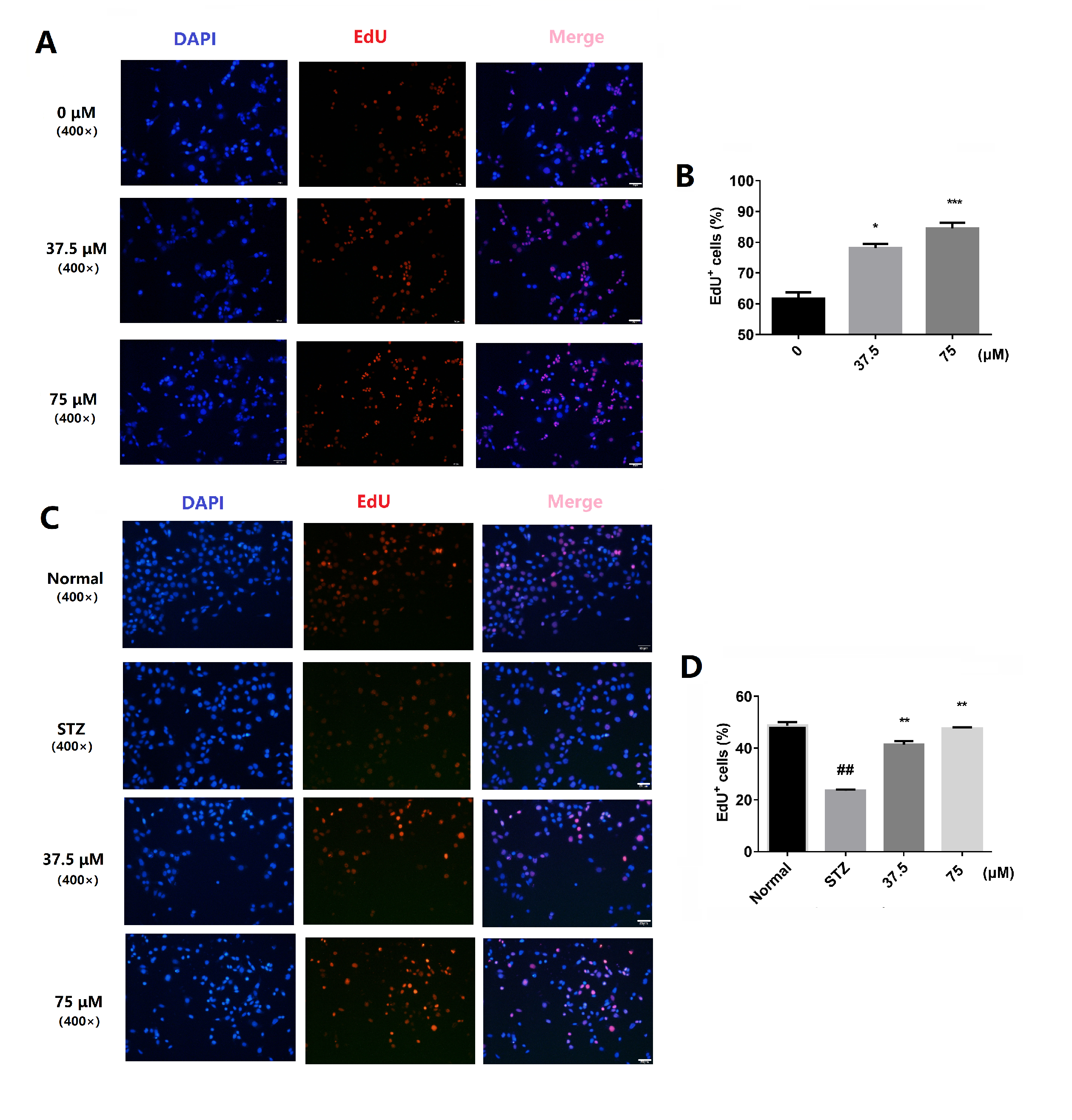

Supplement: Supplementary file 2 — Figure S2 [file CTM2-11-e494-s001.tif]

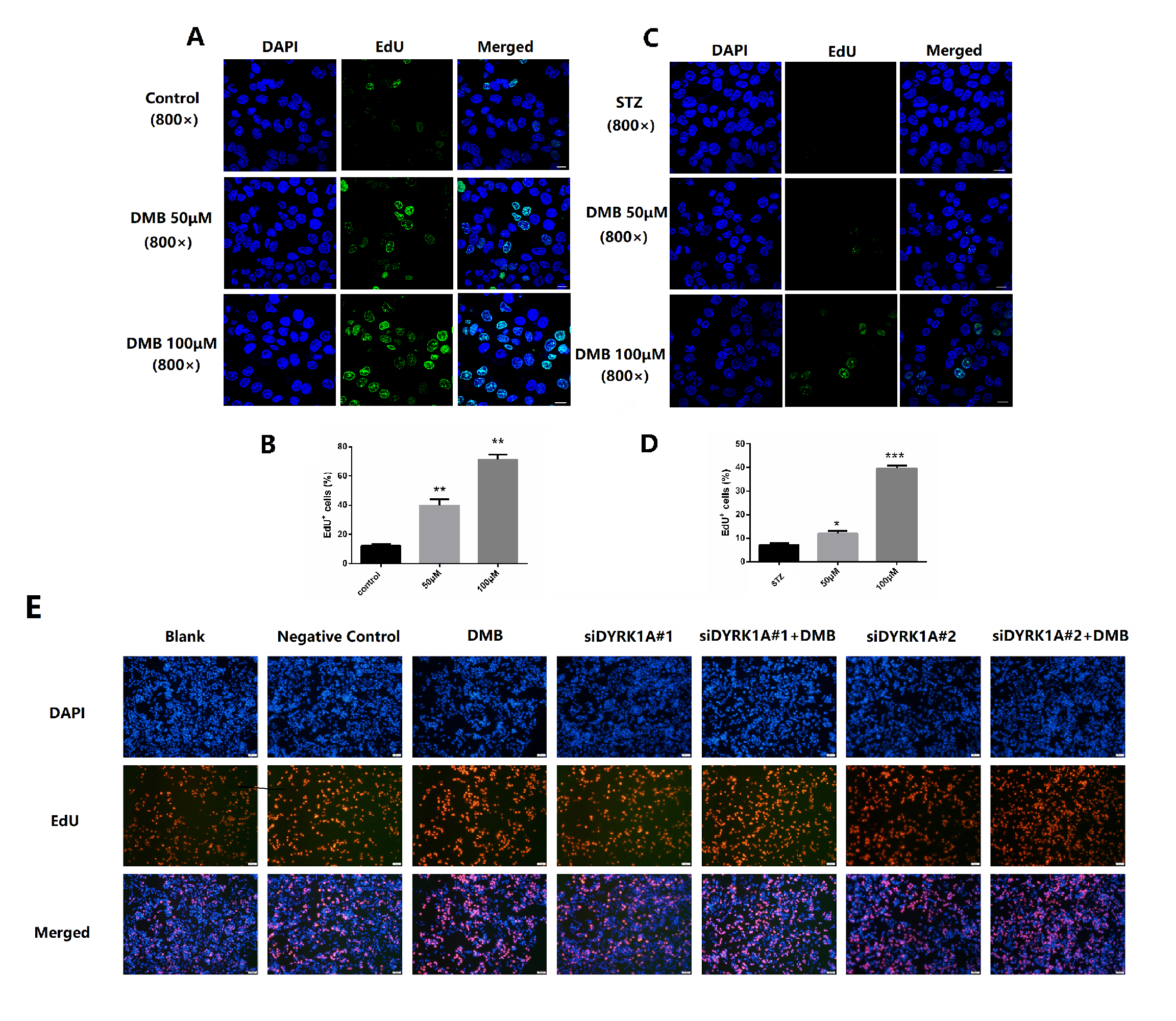

Supplement: Supplementary file 3 — Figure S3 [file CTM2-11-e494-s006.tif]

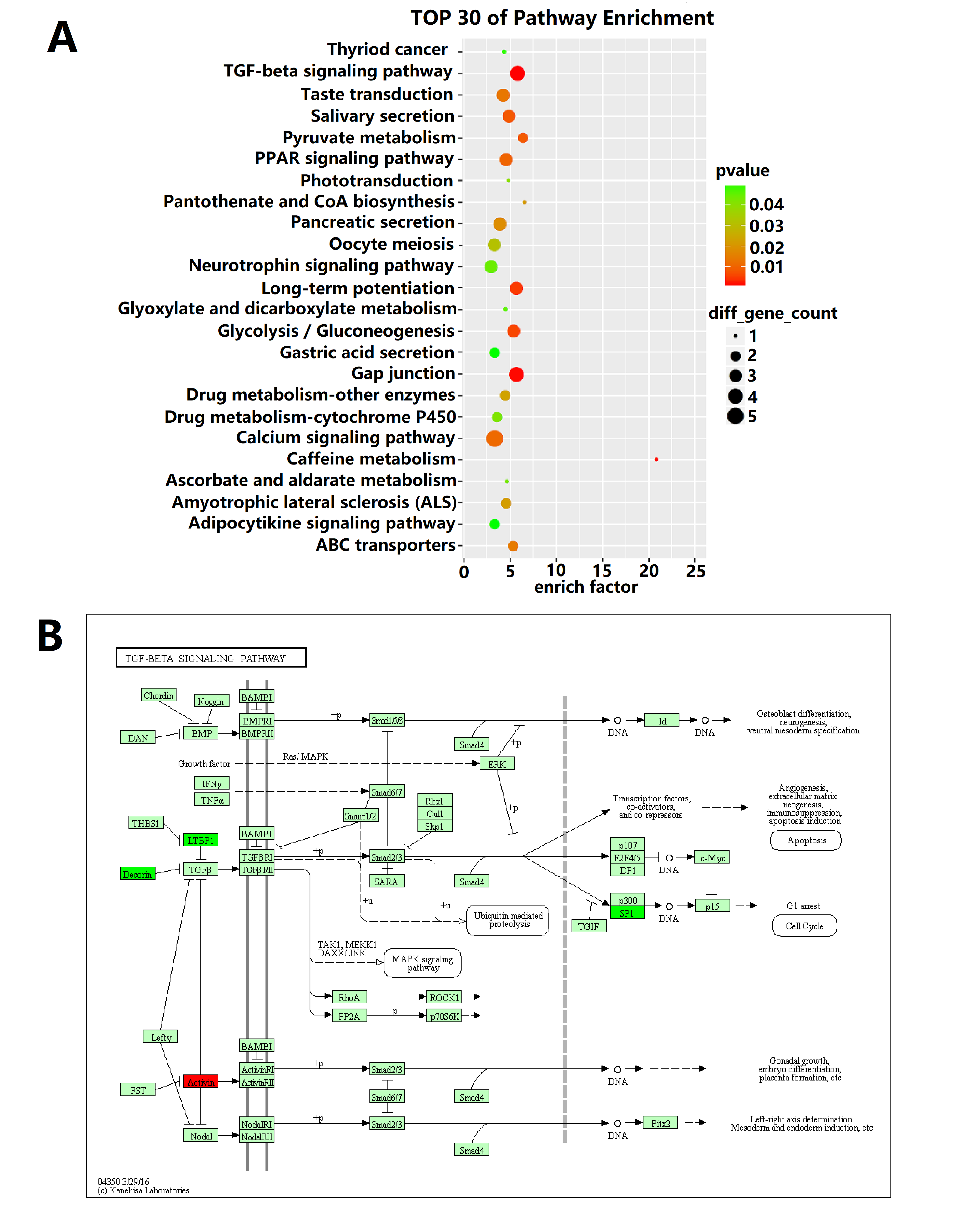

Supplement: Supplementary file 4 — Figure S4 [file CTM2-11-e494-s002.tif]

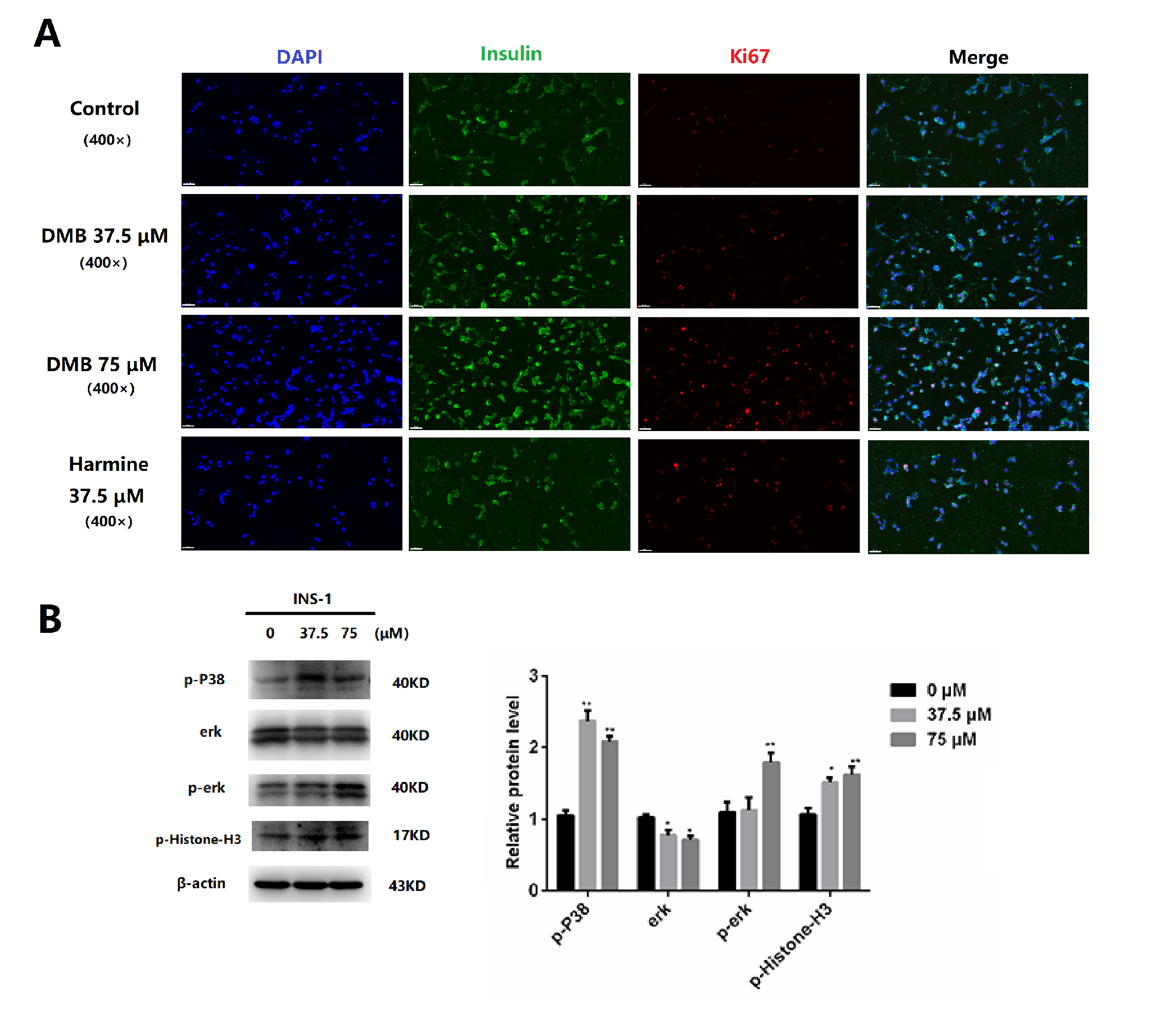

Supplement: Supplementary file 5 — Figure S5 [file CTM2-11-e494-s010.tif]

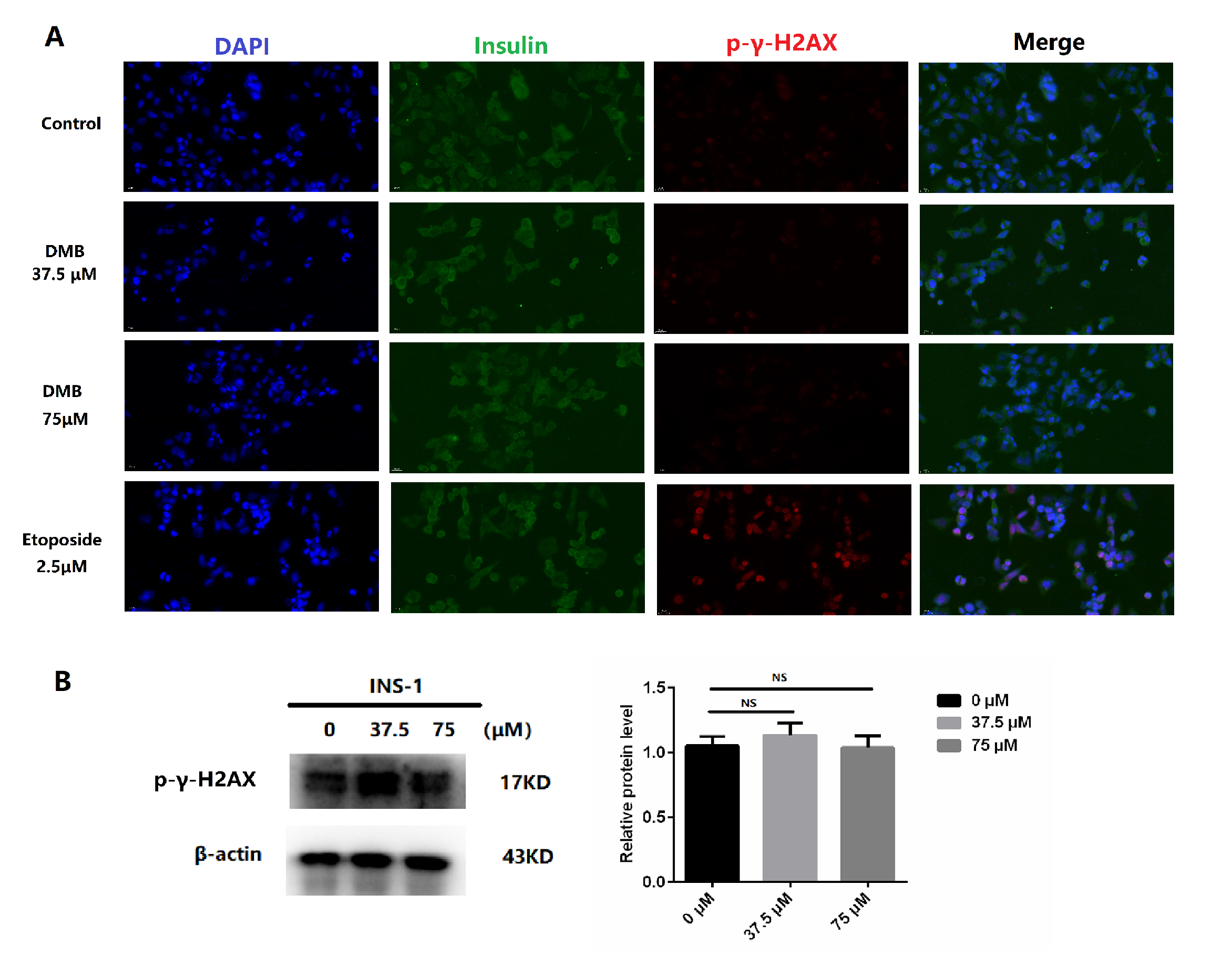

Supplement: Supplementary file 6 — Figure S6 [file CTM2-11-e494-s009.tif]

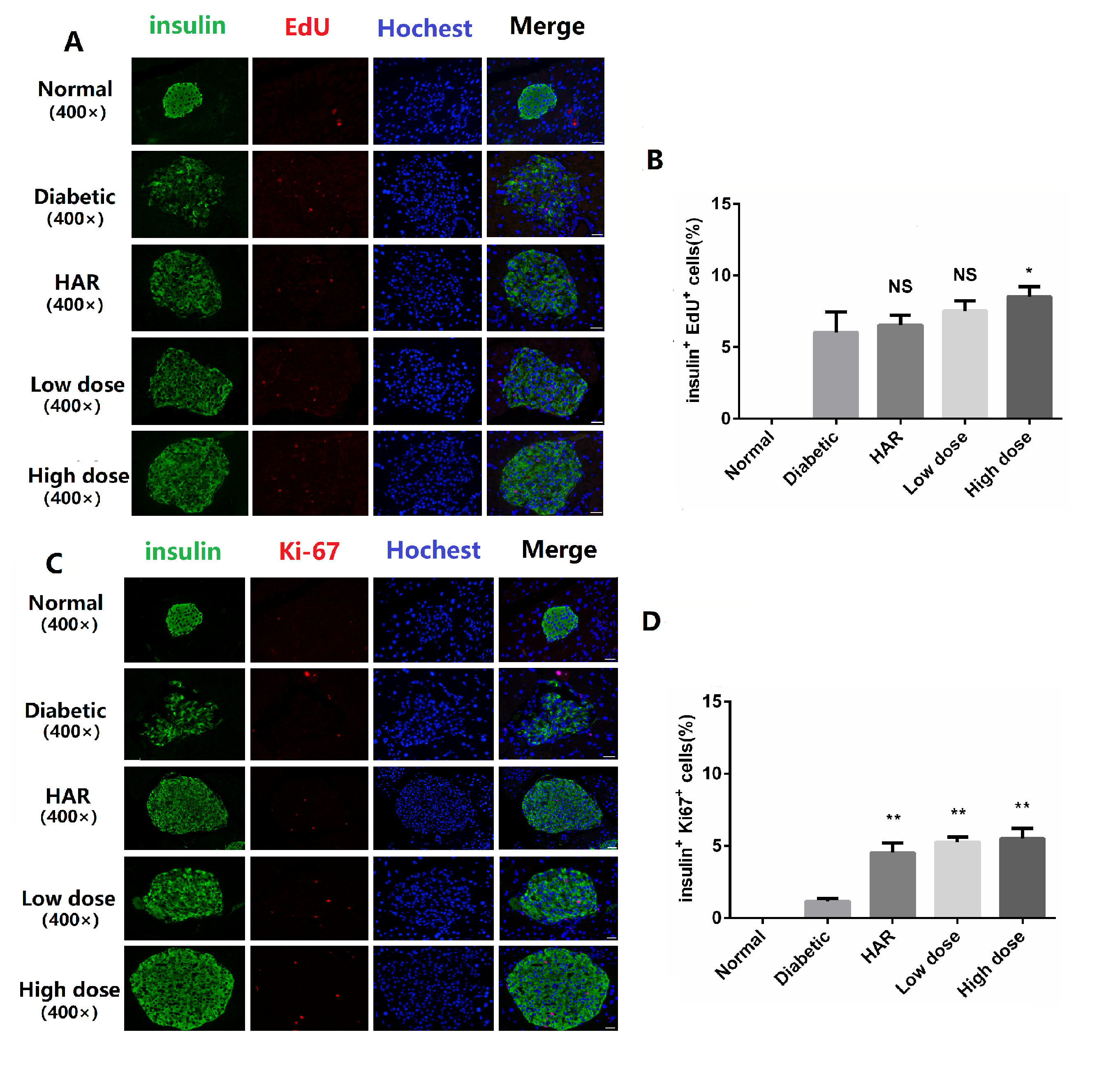

Supplement: Supplementary file 7 — Figure S7 [file CTM2-11-e494-s003.tif]

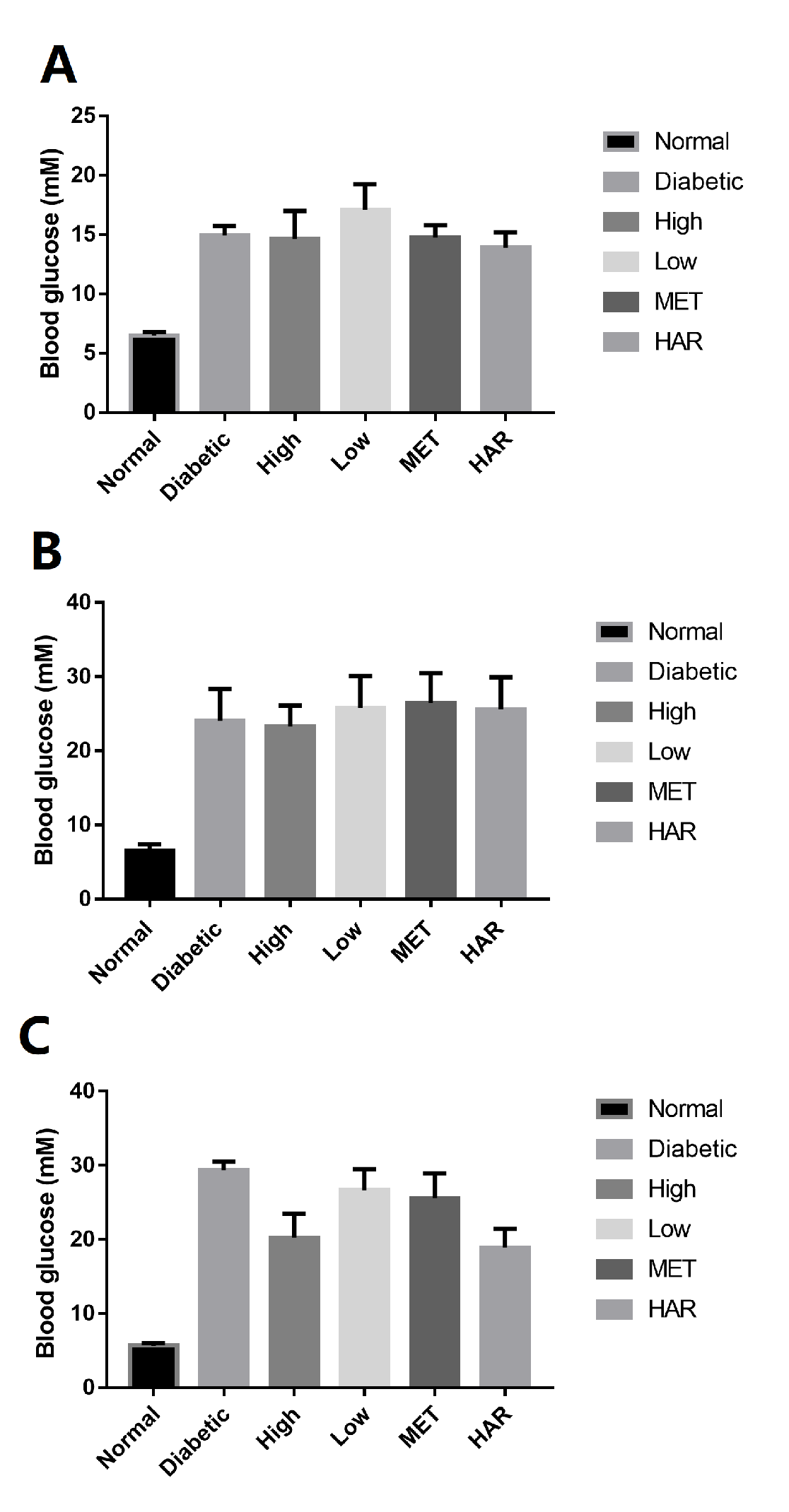

Supplement: Supplementary file 8 — Figure S8 [file CTM2-11-e494-s008.tif]

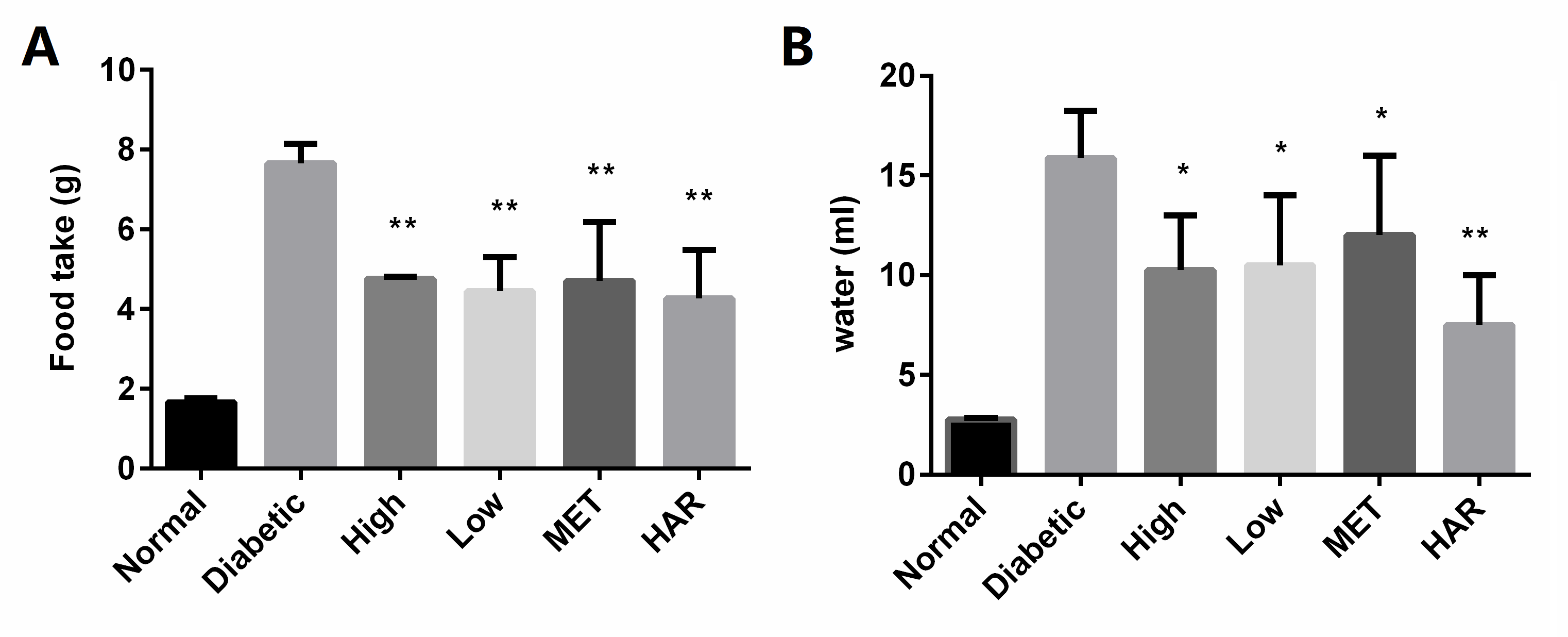

Supplement: Supplementary file 9 — Figure S9 [file CTM2-11-e494-s013.tif]

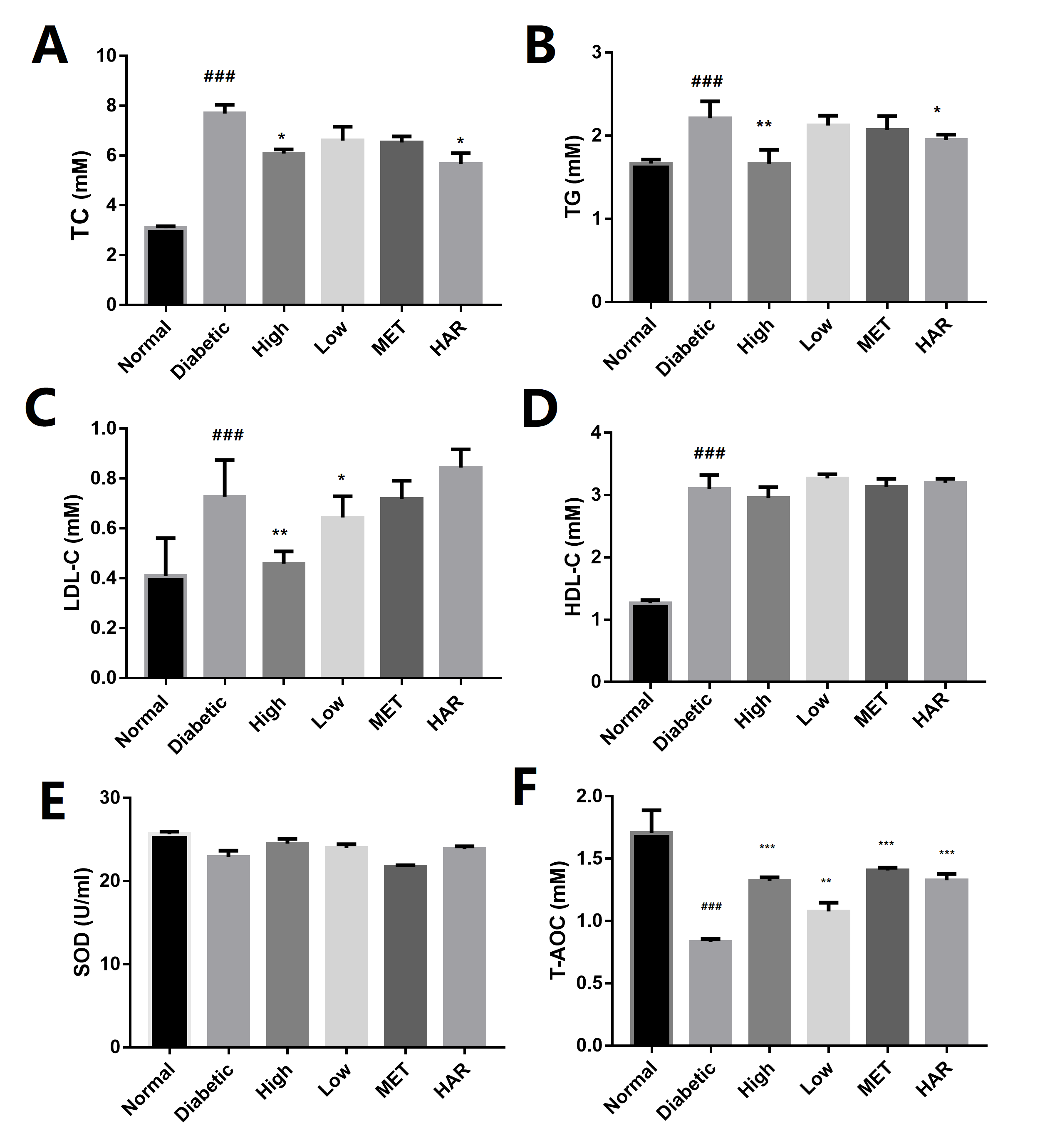

Supplement: Supplementary file 10 — Figure S10 [file CTM2-11-e494-s007.tif]
