# Supplementary material for: A natural DYRK1A inhibitor as a potential stimulator for β‐cell proliferation in diabetes
Source: Clin Transl Med. 2021 Jul 19;11(7):e494. doi: 10.1002/ctm2.494 (PMC8288015; doi:10.1002/ctm2.494)
Supplement: Supplementary file 11 — Supporting Information 1 [file CTM2-11-e494-s005.docx]

**Supporting Information 1:** Supplementary tables

**A natural DYRK1A inhibitor as a potential stimulator for β-cell proliferation in diabetes**

Mengzhu Zheng^1^*, Qingzhe Zhang^1^*, Chengliang Zhang^1,3^*, Canrong Wu^1^, Kaiyin Yang^1^, Zhuorui Song^2^, Qiqi Wang^2^, Chen Li^2^, Yirong Zhou^1^, Jiachun Chen^1†^, Hua Li^1,2†^, Lixia Chen^2†^

**Running title:** DMB can stimulate β-cell proliferation via DYRK1A.

**Affiliations**

^1^ Hubei Key Laboratory of Natural Medicinal Chemistry and Resource Evaluation, School of Pharmacy, Tongji-Rongcheng Center for Biomedicine, Tongji Medical College, Huazhong University of Science and Technology, Wuhan, 430030, China

^2^ Wuya College of Innovation, Key Laboratory of Structure-Based Drug Design & Discovery, Ministry of Education, Shenyang Pharmaceutical University, Shenyang, 110016, China

^3^ Department of Pharmacy, Tongji Hospital, Tongji Medical College, Huazhong University of Science and Technology, Wuhan, 430030, China

* These authors contributed equally to this work.

† Correspondence and requests for materials should be addressed to Lixia Chen (email: [syzyclx@163.com](mailto:syzyclx@163.com)), Hua Li (email: [li_hua@hust.edu.cn](mailto:li_hua@hust.edu.cn)), Jiachun Chen ([homespringchen@126.com](mailto:homespringchen@126.com)).

Hubei Key Laboratory of Natural Medicinal Chemistry and Resource Evaluation, School of Pharmacy, Tongji-Rongcheng Center for Biomedicine, Tongji Medical College, Huazhong University of Science and Technology, Wuhan 430030, P. R. China

Tel: +86-27-83692762 Fax: +86-27-83692762

**S****upplementary tables**

**Table S1. Binding affinities and inhibitory activities of the top-12 screening hits.**

| Cmpd | ICM docking Scores^a^  (kcal/mol) | ICM docking mfScores^b^  (kcal/mol) | Inhibitory activities against DYRK1A enzyme | Dissociation constant with DYRK1A |
| --- | --- | --- | --- | --- |
|  |  |  | IC_50_ (μM) | K_d_^c^ (μM) |
| **1** | -33.00 | -112.18 | 0.37 ± 0.01 | 5.11 ± 0.33 |
| **2** | -32.87 | -143.87 | 0.74 ± 0.17 | 93.50 ± 31.50 |
| **3** | -27.58 | -137.15 | 5.44 ± 0.67 | 373.00 ± 59.70 |
| **4** | -28.05 | -112.46 | 16.78 ± 1.78 | 130.00 ± 19.40 |
| **5** | -24.04 | -138.41 | > 250 | 558.00 ± 74.30 |
| **6** | -34.24 | -151.29 | 174.20 ± 18.35 | 348.00 ± 65.50 |
| **7** | -24.80 | -141.92 | > 250 | 473.00 ± 141.00 |
| **8** | -16.39 | -139.35 | > 250 | 441.00 ± 155.00 |
| **9** | -26.10 | -130.83 | 49.15 ± 1.13 | ^e^n.b. |
| **10** | -31.93 | -137.09 | > 250 | ^e^n.b. |
| **11** | -18.63 | -133.70 | > 250 | ^e^n.b. |
| **12** | -18.17 | -127.19 | > 250 | 408.00 ± 135.00 |
| **Harmine** | n.p^d^ | n.p^d^ | 2.51 ± 0.13 | 81.70 ± 12.80 |

^a^Docking score/interaction potential of compounds with DYRK1A (kcal/mol).

^b^Docking mfScore/interaction potential of compounds with DYRK1A (kcal/mol).

^c^The K_d_ value is automatic calculated by the curve fitting, and presents as means ± SD, n = 3.

^d^n.p. was not performed in this study.

^e^n.b. means no binding.

**Table S2. Crystallographic Data and Refinement Statistics**

| Parameters | DMB/Dyrk1A complex |
| --- | --- |
| **Data collection** |  |
| Space group | P63 |
| Cell dimensions |  |
| a, b, c (Å) | 133.467 133.467 92.238 |
| α, β, γ (°) | 90, 90, 120 |
| Resolution range (Å) | 48.97-2.50(2.75-2.70) |
| Rsym (%) | 1.1 (38.4) |
| Completeness (%)*^a^* | 99.9 (99.9) |
| Redundancy | 9.8 (10.6) |
| *I/σI^a^* | 68.98 (1.52) |
| **Refinement** |  |
| Total reflections | 25854 |
| *R*_work_/*R*_free_^c^ | 0.212,0.277 |
| No. atoms |  |
| Protein | 5528 |
| Ligand | 54 |
| Water | 50 |
| Average *B*-factor (Å2) | 65.0 |
| Ramachandran Plot (%) |  |
| Favored | 91.83 |
| Allowed | 7.41 |
| Outliers | 0.76 |
| Root mean square deviations |  |
| R.m.s.d. bond lengths (Å) | 0.119 |
| R.m.s.d. bond angle (°) | 4.24 |

Values in parenthesis are for highest resolution shell. 5 % of the data was used in the Rfree calculation.

**Table S3. Comparison of distance between atoms that may form hydrogen bonds in DMB docking model and crystal structure**

| Groups in DMB | Residues of Dyrk1A | Distance between atoms that may form hydrogen bonds | | | |  |
| --- | --- | --- | --- | --- | --- | --- |
|  |  | In docking structure (Å) | | In co-crystal structure (Å) | | |
| 8-hydroxyl | Lys 188 | | 3.2 | 3.0 | | |
| 1-hydroxyl | Glu 239 | | 2.6 | 4.3 | | |
| 3-hydroxyl | Leu 241 | | 2.9 | 2.0 | | |
| **Table S4. KEGG pathway enrichment by Gene Expression Analysis.**   \| Description \| listUp \| listDown \| \| --- \| --- \| --- \| \| Caffeine metabolism \| *NAT1* \|  \| \| Pantothenate and CoA biosynthesis \|  \| *PANK2* \| \| Pyruvate metabolism \| *PCK1 LDHAL6B* \|  \| \| TGF-beta signaling pathway \| *INHBE* \| *DCN LTBP1 SP1* \| \| Gap junction \| *GRM1* \| *TJP1 ADCY6 HTR2C* \| \| Long-term potentiation \| *GRIN2A GRM1* \| *RPS6KA3* \| \| Glycolysis / Gluconeogenesis \| *PCK1 PGK1 LDHAL6B* \|  \| \| ABC transporters \| *ABCG1 ABCA8A* \|  \| \| Salivary secretion \| *KCNN4 CD38* \| *ADCY6* \| \| Phototransduction \|  \| *RHO* \| \| Ascorbate and aldarate metabolism \| *UGT2B7* \|  \| \| PPAR signaling pathway \| *PLTP FABP7 PCK1* \|  \| \| Amyotrophic lateral sclerosis (ALS) \| *GRIN2A TOMM40L* \|  \| \| Glyoxylate and dicarboxylate metabolism \| *AFMID* \|  \| \| Drug metabolism - other enzymes \| *NAT1 UGT2B7* \|  \| \| Thyroid cancer \|  \| *PAX8* \| \| Taste transduction \| *TAS2R126 GRM1* \| *ADCY6* \| \| Circadian rhythm \|  \| *BTRC* \| \| Nicotinate and nicotinamide metabolism \| *CD38* \|  \| \| Propanoate metabolism \| *LDHAL6B* \|  \| \| Citrate cycle (TCA cycle) \| *PCK1* \|  \| \| Pancreatic secretion \| *CD38 ATP2A3* \| *ADCY6* \| \| Prion diseases \|  \| *NCAM1* \| \| Drug metabolism - cytochrome P450 \| *FMO5 UGT2B7* \|  \| \| Pentose and glucuronate interconversions \| *UGT2B7* \|  \| \| Gastric acid secretion \| *KCNE2* \| *ADCY6* \| \| Calcium signaling pathway \| *GRIN2A CD38 GRM1 ATP2A3* \| *HTR2C* \| \| Adipocytokine signaling pathway \| *PCK1* \| *IRS1* \| \| Primary immunodeficiency \|  \| *JAK3* \| \| Oocyte meiosis \|  \| *RPS6KA3 BTRC ADCY6* \| \| Porphyrin and chlorophyll metabolism \| *UGT2B7* \|  \| \| Aldosterone-regulated sodium reabsorption \|  \| *IRS1* \| \| Retinol metabolism \| *UGT2B7* \| *ALDH1A1* \| \| Neurotrophin signaling pathway \| *NTRK3* \| *RPS6KA3 IRS1* \| \| Vasopressin-regulated water reabsorption \|  \| *ADCY6* \| \| Hedgehog signaling pathway \|  \| *BTRC* \| \| Progesterone-mediated oocyte maturation \|  \| *RPS6KA3 ADCY6* \| \| Protein digestion and absorption \| *KCNN4* \| *COL5A3* \| \| Tryptophan metabolism \| *AFMID* \|  \| \| Cysteine and methionine metabolism \| *LDHAL6B* \|  \| \| Tight junction \| *ASH1L CLDN11* \| *TJP1* \| \| Melanogenesis \|  \| *ADCY6 TYRP1* \| \| Type II diabetes mellitus \|  \| *IRS1* \| \| Cell adhesion molecules (CAMs) \| *NRCAM CLDN11* \| *NCAM1* \| \| Lysine degradation \| *ASH1L* \|  \| \| Leukocyte transendothelial migration \| *TXK CLDN11* \|  \| \| Glutathione metabolism \| *GGCT* \|  \| \| Long-term depression \| *GRM1* \|  \| \| Graft-versus-host disease \| *KLRD1* \|  \| \| Metabolism of xenobiotics by cytochrome P450 \| *UGT2B7* \|  \| \| Aminoacyl-tRNA biosynthesis \|  \| *FARS2* \| \| Ubiquitin mediated proteolysis \|  \| *BTRC HERC4* \| \| Insulin signaling pathway \| *PCK1* \| *IRS1* \| \| Melanoma \| *FGF13* \|  \| \| Bile secretion \|  \| *ADCY6* \| \| Neuroactive ligand-receptor interaction \| *GRIN2A GRM1 GHSR* \| *HTR2C* \| \| Adherens junction \|  \| *TJP1* \| \| mTOR signaling pathway \|  \| *RPS6KA3 IRS1* \| \| Steroid hormone biosynthesis \| *UGT2B7* \|  \| \| Bacterial invasion of epithelial cells \| *SEPT3* \|  \| \| Cardiac muscle contraction \| *MYL4* \|  \| \| Complement and coagulation cascades \| *SERPINB2* \|  \| \| ECM-receptor interaction \|  \| *LAMC2* \| \| Small cell lung cancer \|  \| *LAMC2* \| \| Rheumatoid arthritis \|  \| *TNFSF11* \| \| Dilated cardiomyopathy \|  \| *ADCY6* \| \| Chemokine signaling pathway \|  \| *JAK3 ADCY6* \| \| Axon guidance \| *SEMA6C EFNA5* \|  \| \| Alzheimer's disease \| *GRIN2A ATP2A3* \|  \| \| GnRH signaling pathway \|  \| *ADCY6* \| \| Hematopoietic cell lineage \| *CD38* \|  \| \| mRNA surveillance pathway \|  \| *MSI2* \| \| Toll-like receptor signaling pathway \|  \| *TLR1* \| \| Antigen processing and presentation \| *KLRD1* \|  \| \| Natural killer cell mediated cytotoxicity \| *KLRD1* \|  \| \| Pathways in cancer \| *FGF13* \| *LAMC2 PAX8 ADCY6* \| \| Huntington's disease \|  \| *DLG4 SP1* \| \| Amoebiasis \|  \| *LAMC2* \| \| Toxoplasmosis \|  \| *LAMC2* \| \| Vascular smooth muscle contraction \|  \| *ADCY6* \| \| Systemic lupus erythematosus \| *GRIN2A* \|  \| \| MAPK signaling pathway \| *FGF13* \| *RPS6KA3* \| \| Hepatitis C \| *CLDN11* \|  \| \| Osteoclast differentiation \|  \| *TNFSF11* \| \| Wnt signaling pathway \|  \| *BTRC* \| \| Jak-STAT signaling pathway \|  \| *JAK3* \| \| Purine metabolism \|  \| *ADCY6* \| \| Focal adhesion \|  \| *LAMC2* \| \| Cytokine-cytokine receptor interaction \|  \| *TNFSF11* \| \| Regulation of actin cytoskeleton \| *FGF13* \|  \| \| Endocytosis \|  \| *SH3KBP1* \| \| Olfactory transduction \|  \| *OLR1654* \| | | | | |  |  |

**Table S5. Effects of drugs on serum GSH levels in mice.**

| Group | GSH (μM) |
| --- | --- |
| Normal | 10.31 ± 6.62 |
| Diabetic | 11.81 ± 9.00^##^ |
| Metformin | 13.47 ± 8.09 |
| Harmine | 11.53 ± 8.71 |
| Low-dose DMB | 15.00 ± 6.06 |
| High-dose DMB | 23.07 ± 9.22^*^ |

Each value is expressed as the mean ± SD, n = 8; Metformin (200 mg/kg) and harmine (200 mg/kg) were used as positive controls. ^#^*p* < 0.05, ^##^*p* < 0.01, compared to the normal group; ^*^*p* < 0.05, ^**^*p* < 0.01, compared to the diabetic group.
