# Supplementary material for: A natural DYRK1A inhibitor as a potential stimulator for β‐cell proliferation in diabetes
Source: Clin Transl Med. 2021 Jul 19;11(7):e494. doi: 10.1002/ctm2.494 (PMC8288015; doi:10.1002/ctm2.494)
Supplement: Supplementary file 12 — Supporting Information 2 [file CTM2-11-e494-s011.docx]

**Supporting Information 2:** Supplementary Figures

**A natural DYRK1A inhibitor as a potential stimulator for β-cell proliferation in diabetes**

Mengzhu Zheng^1^*, Qingzhe Zhang^1^*, Chengliang Zhang^1,3^*, Canrong Wu^1^, Kaiyin Yang^1^, Zhuorui Song^2^, Qiqi Wang^2^, Chen Li^2^, Yirong Zhou^1^, Jiachun Chen^1†^, Hua Li^1,2†^, Lixia Chen^2†^

**Running title:** DMB can stimulate β-cell proliferation via DYRK1A.

**Affiliations**

^1^ Hubei Key Laboratory of Natural Medicinal Chemistry and Resource Evaluation, School of Pharmacy, Tongji-Rongcheng Center for Biomedicine, Tongji Medical College, Huazhong University of Science and Technology, Wuhan, 430030, China

^2^ Wuya College of Innovation, Key Laboratory of Structure-Based Drug Design & Discovery, Ministry of Education, Shenyang Pharmaceutical University, Shenyang, 110016, China

^3^ Department of Pharmacy, Tongji Hospital, Tongji Medical College, Huazhong University of Science and Technology, Wuhan, 430030, China

* These authors contributed equally to this work.

† Correspondence and requests for materials should be addressed to Lixia Chen (email: [syzyclx@163.com](mailto:syzyclx@163.com)), Hua Li (email: [li_hua@hust.edu.cn](mailto:li_hua@hust.edu.cn)), Jiachun Chen ([homespringchen@126.com](mailto:homespringchen@126.com)).

Hubei Key Laboratory of Natural Medicinal Chemistry and Resource Evaluation, School of Pharmacy, Tongji-Rongcheng Center for Biomedicine, Tongji Medical College, Huazhong University of Science and Technology, Wuhan 430030, P. R. China

Tel: +86-27-83692762 Fax: +86-27-83692762

**Supplementary Figures**


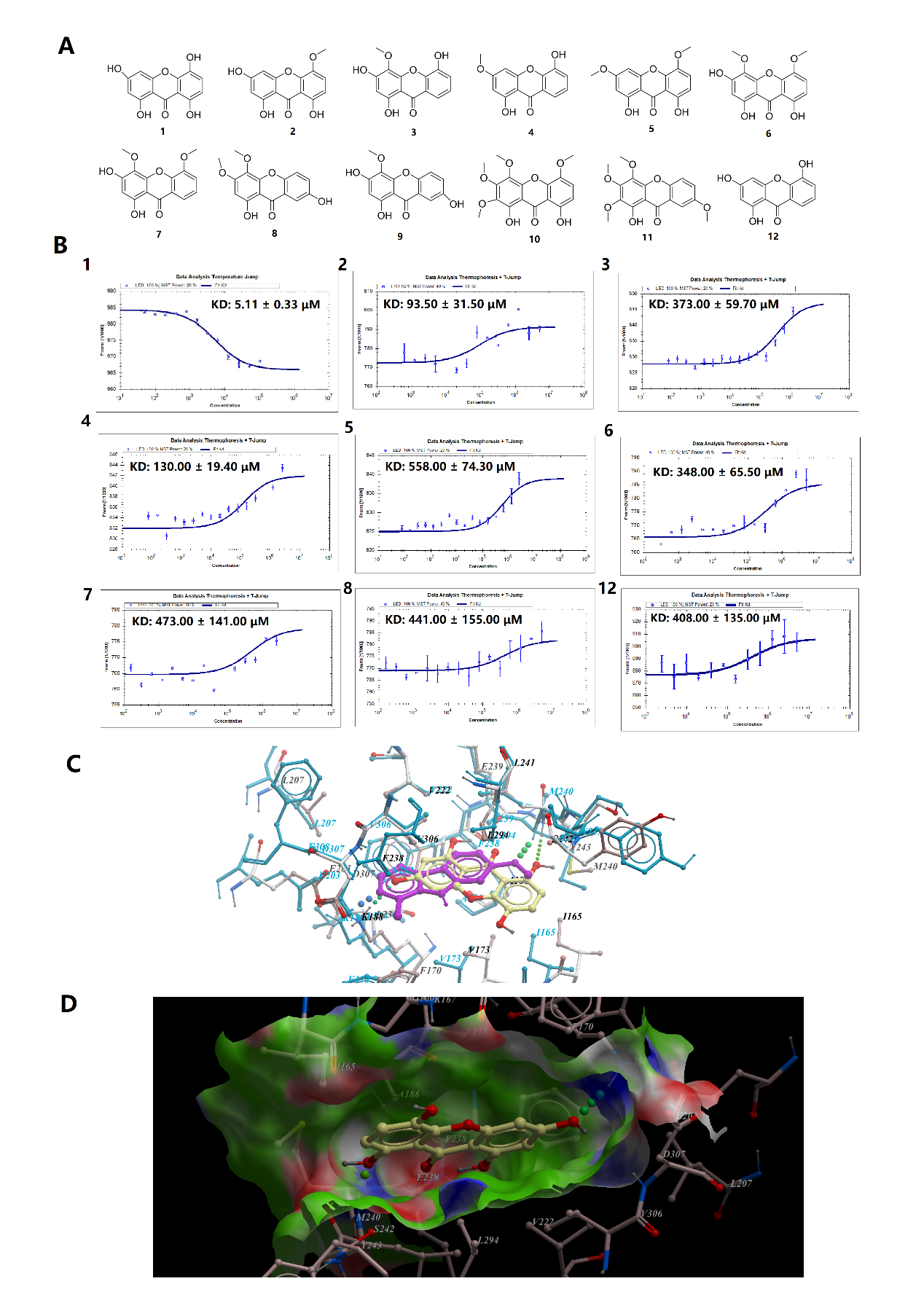


**Figure S1 The binding affinities of xanthones with DYRK1A and the binding mode of DMB with DYRK1A. (A)** Chemical structures of the top twelve xanthones (**1-12**) selected from the databank and evaluated in this study. **(B)** Binding affinity of compounds **1-12** with DYRK1A. Among the 12 natural candidates tested, DMB exhibited the strongest binding with DYRK1A. **(C)** Overlay of DYRK1A-DMB binary complex. DMB was docked to DYRK1A by using ICM-Pro 3.8.1 software. It was found to adopt an extended conformation, which almost completely occupied the whole flat-shaped pocket. **(D)** Detailed view of DMB and harmine binding in the ligand binding pocket. DMB adopted a similar conformation as that of harmine (PDB ID 3ANR), translated approximately 30 degrees, and shifted a distance of a half ring. Similar to that of DMB, harmine also formed two hydrogen bonds with Lys188 and Leu241, and its pyrrole ring interacted with Val173, Leu294, Val306, and Phe238.


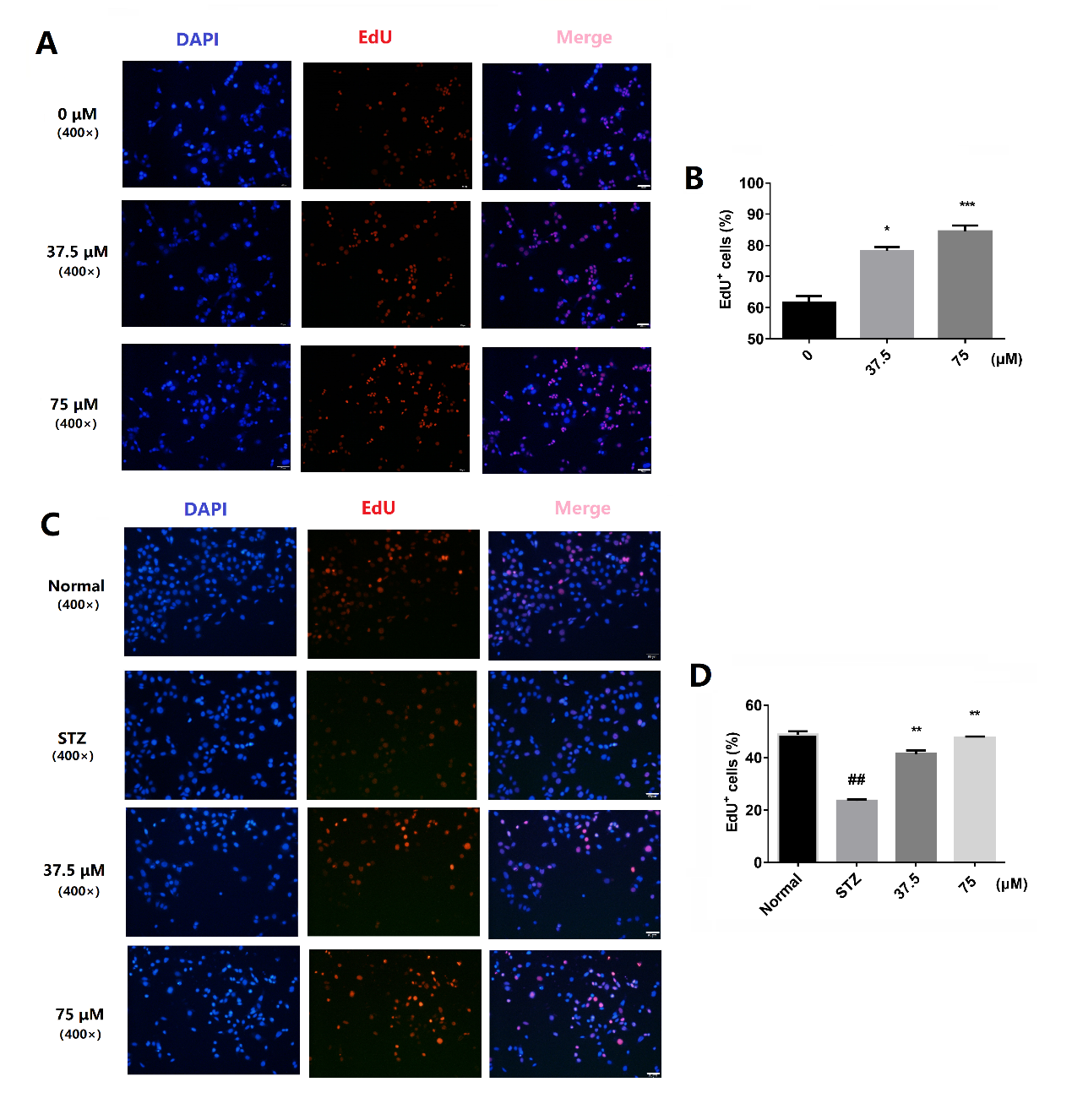


**Figure S2 DMB promoted INS-1 cell proliferation and regeneration (A)** Representative images of the effects of DMB (37.5 µM and 75 µM) on INS-1 cells which were stained with EdU (red) and DAPI (blue) (Magnification 400 ×, the scale bar indicates 50 μm). **(B)** The percentage of new EdU^+^-INS-1 cells after DMB administration (n = 3). (**p* < 0.05, ***p* < 0.01, ****p* <0.001). **(C)** Representative images of the effects of DMB (37.5 µM and 75 µM) on INS-1 cells in STZ-induced cell damage model which were stained with EdU (red) and DAPI (blue) (Magnification 400 ×, the scale bar indicates 50 μm). **(D)** The percentage of new EdU^+^-INS-1 cells in STZ-induced cell damage model after DMB administration (n = 3). (**p* < 0.05, ***p* < 0.01, ****p* <0.001, ^##^*p* ≤ 0.01). The proliferation percentages were 23.0%, 42.7% and 47.3% at a concentration of 0, 37.5 and 75 μM of DMB, respectively.


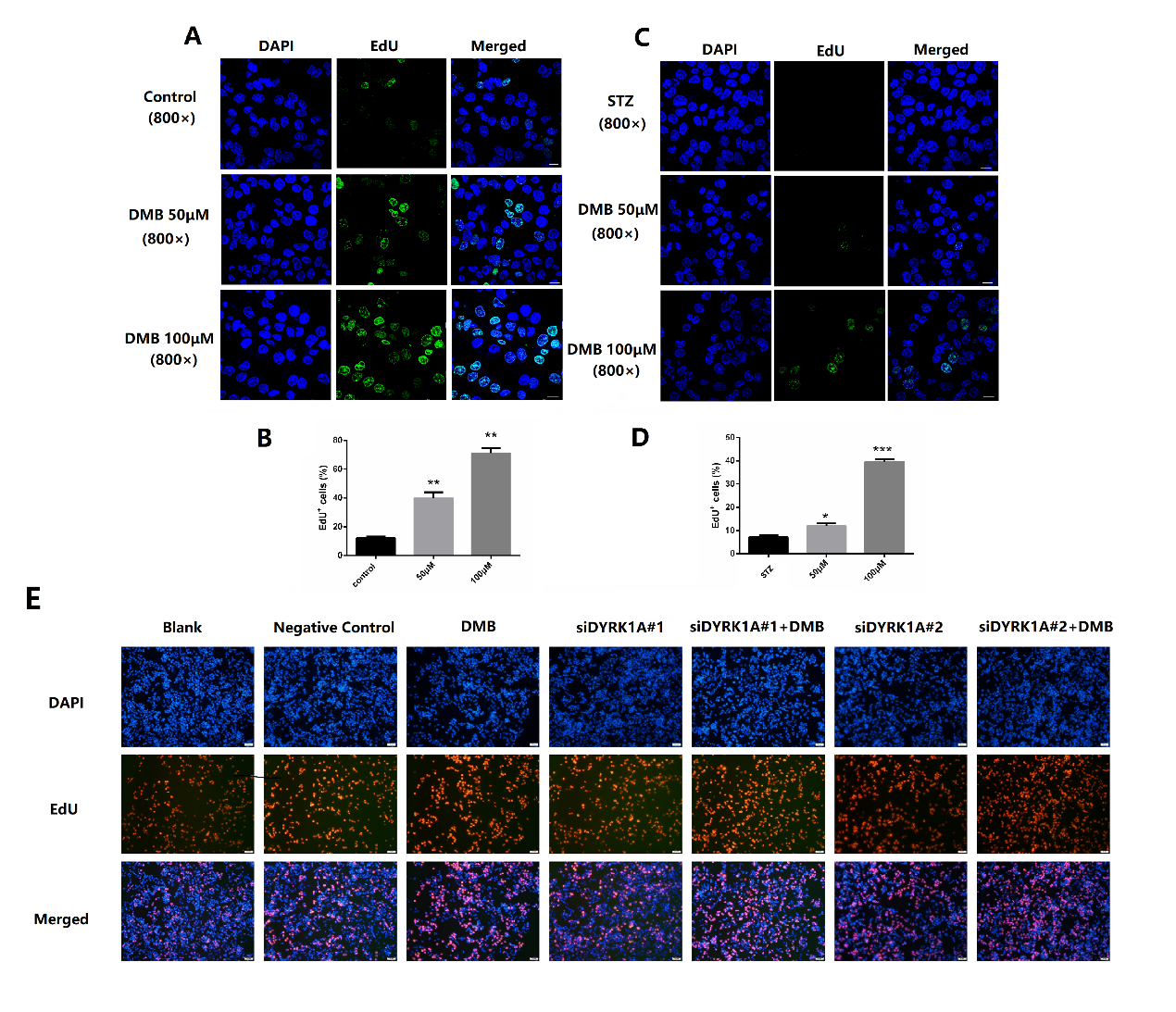


**Figure S3 Representative examples of EdU-labeled β-cells after treatment with DMB in INS-1 normal cells (A, B) and in streptozotocin induced INS-1 damage cells (C, D)**. Double-immunofluorescence staining with EdU (green) and Hoechst (blue) (Magnification 800 ×, the scale bar indicates 20 μm). **(E)** Representative images of the effects of different groups on INS-1 cell stained with EdU (red) and Hoechst (blue). Statistics of EdU fluorescent spots were shown in **Figure 1O**. Compared to those in the blank and negative control groups, siDYTK1A group significantly promoted the proliferation of INS-1 cells. However, additional administration of DMB in siDYRK1A#1 and siDYRK1A#2 groups did not further increase the proliferation of INS-1 cells.


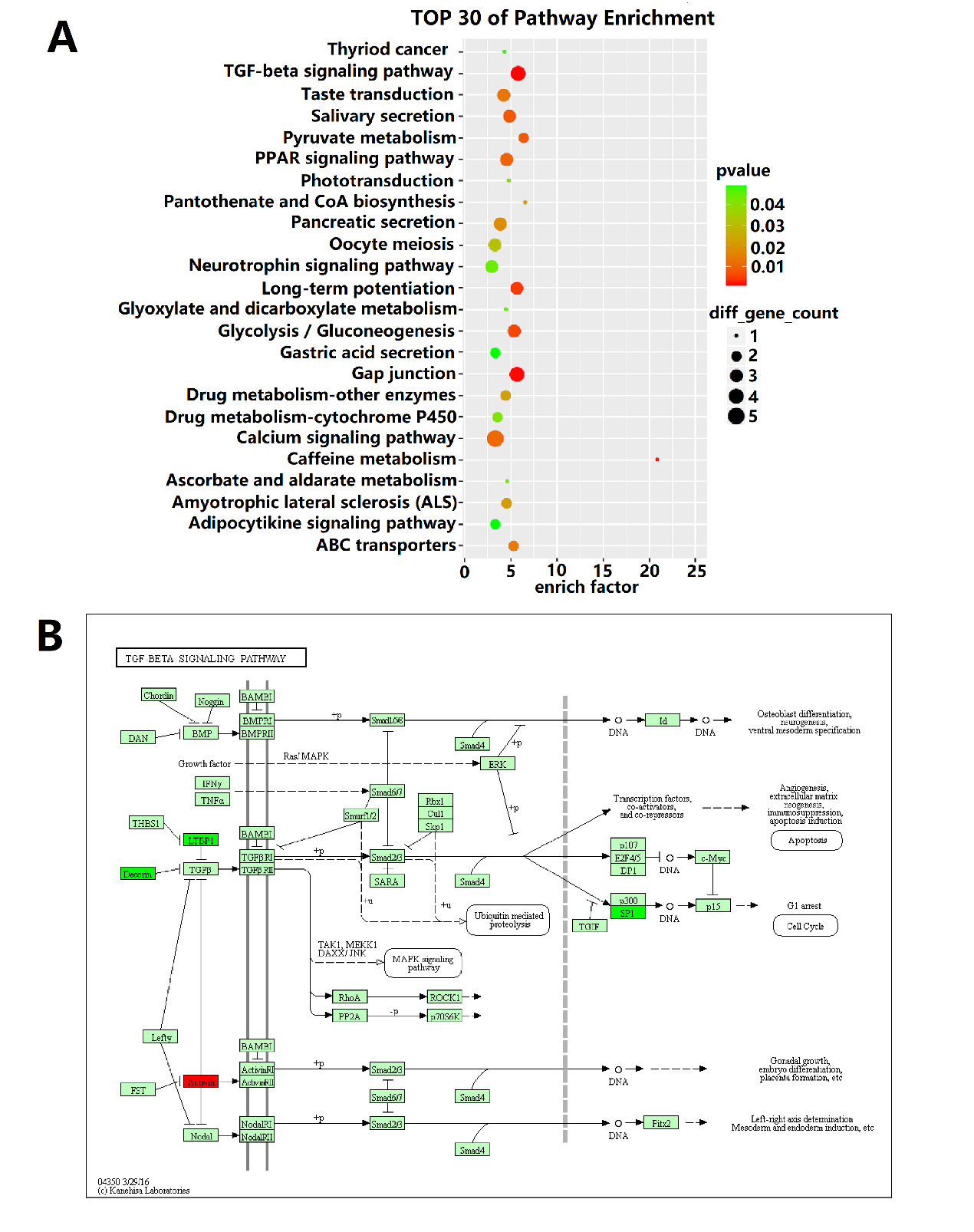


**Figure S4 Gene expression analysis of DMB-treated INS-1 cells. (A)** Top 30 of pathway enrichment by Gene Expression Analysis. **(B)** TGF-β signaling pathway by KEGG (the green node is the down-regulated gene, the red node is the up-regulated gene, and the light green is the presence of the gene in the species).


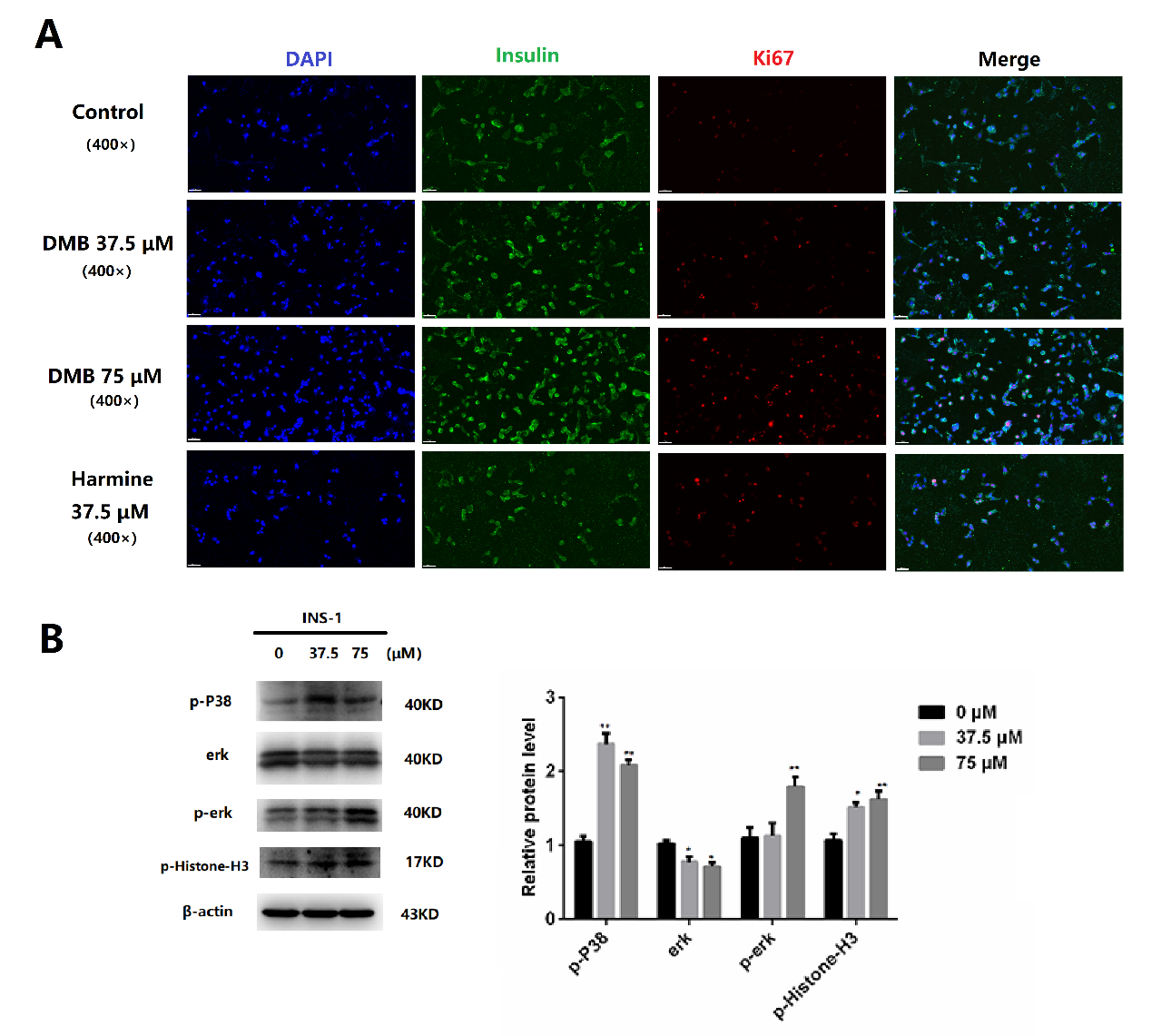


**Figure S5** **Effects of DMB on markers of β-cells proliferation**. (**A**) Representative examples of immunofluorescence for Ki67^+^ and insulin in β-cells treated with vehicle or DMB (Magnification 400 ×, the scale bar indicates 50 μm). DMB and harmine had the comparable promotion effect on INS-1 cell proliferation at the same concentration (37.5 μM). (**B**) Representative examples of Western blot for p-P38, erk, p-erk, insulin, p-Histone-H3 in β-cells treated with DMB as above. Protein levels were quantified using grey value analyses by Image J software in the right. DMB induced phosphorylation of histone H3, a third marker of cell-cycle transition. Proliferation-related indicators, p-P38 and p-erk, were increased at the protein levels in β-cells.


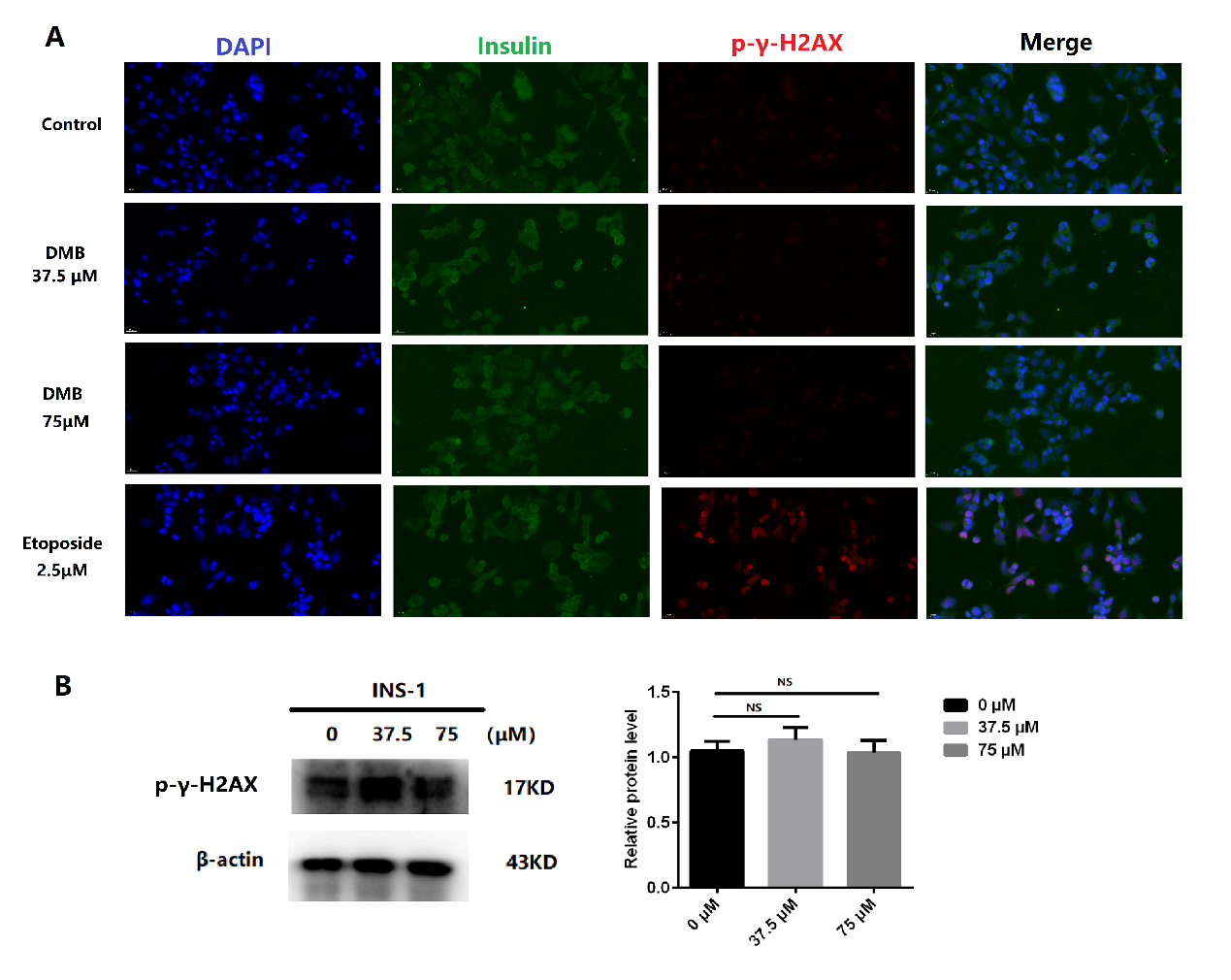


**Figure S6 No evidence of** **β-cell death or DNA damage in response to DMB as measured by p-γ-H2AX labeling. (A)** Representative images of the effects of DMB (37.5 µM and 75 µM) treatment on p-γ-H2AX of INS-1 cells (Magnification 400 ×, the scale bar indicates 50 μm). The inset in the etoposide panel includes INS-1 cells that were used as positive control. **(B)** Representative Western blot of p-γ-H2AX in INS-1 cells treated with DMB.


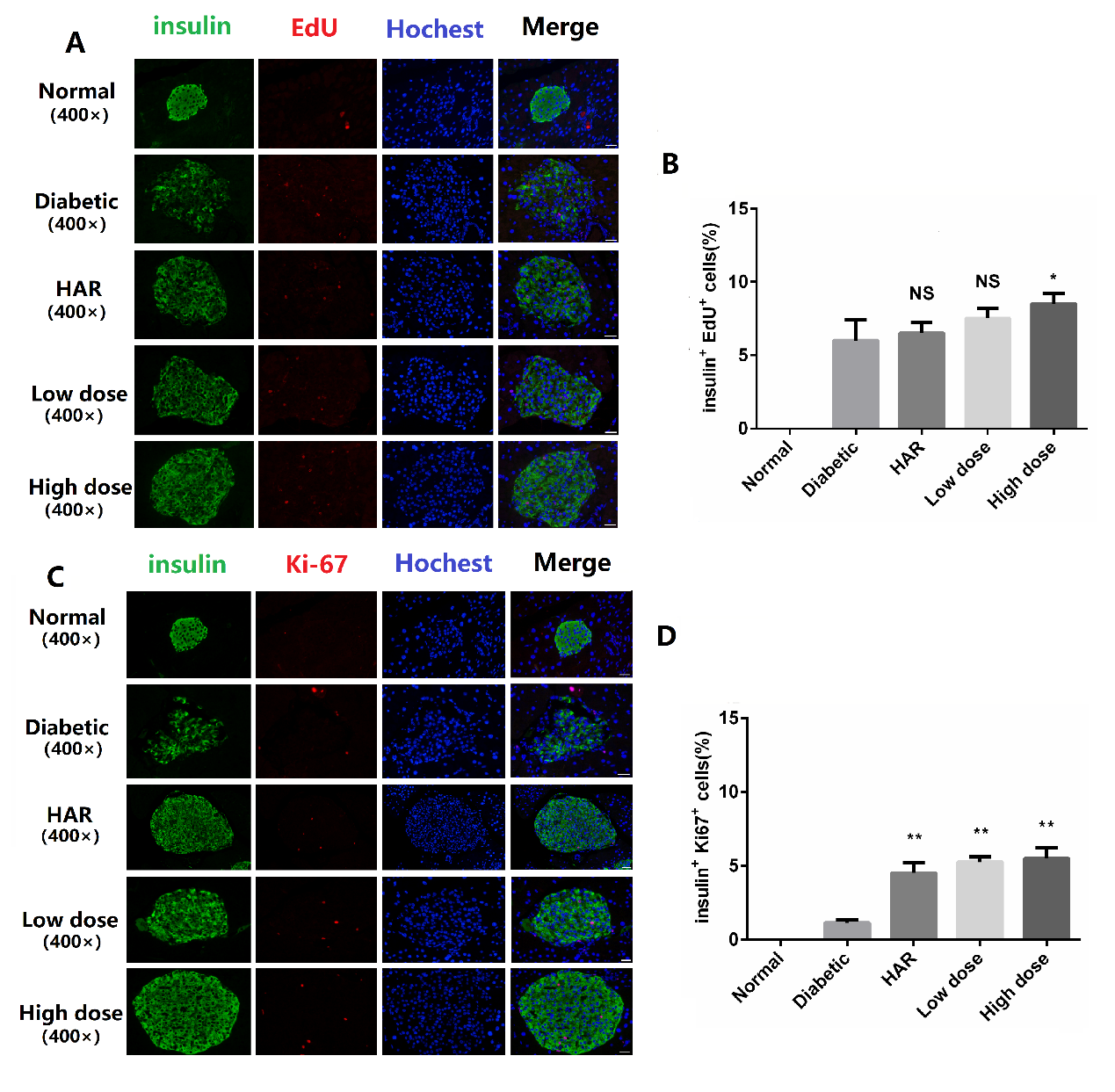


**Figure S7 Immunofluorescence analysis of DMB induced mice β-cell replication and regeneration *in vivo*.** (**A**) Representative immunofluorescence images of EdU-labeled of pancreases β-cell with insulin (green), EdU (red) and Hoechst (blue) after treatment with harmine and DMB (75 mg/kg and 150 mg/kg) (Magnification 400 ×, the scale bar indicates 50 μm). The pancreatic β-cell mass and size were higher in both DMB- and harmine-treated groups, as compared to these parameters in diabetic model group. The β-cell proliferation rate was a little higher in the high-dose DMB-treated group compared to that in diabetic model group. (**B**) The percentage of insulin^+^-EdU^+^ cells after compounds administration compared with control (^*^*p* < 0.05, ^**^*p* < 0.01). (**C**) Representative immunofluorescence images of Ki-67 immunolabeling of pancreases β-cell in the same experiment as in (**A**) (Magnification 400 ×, the scale bar indicates 50 μm). The number of insulin/Ki67 double-positive cells in the DMB-treated group was significantly increased compared to that in the diabetic model group. (**D**) Quantification of Ki-67 immunolabeling in pancreas β-cells.


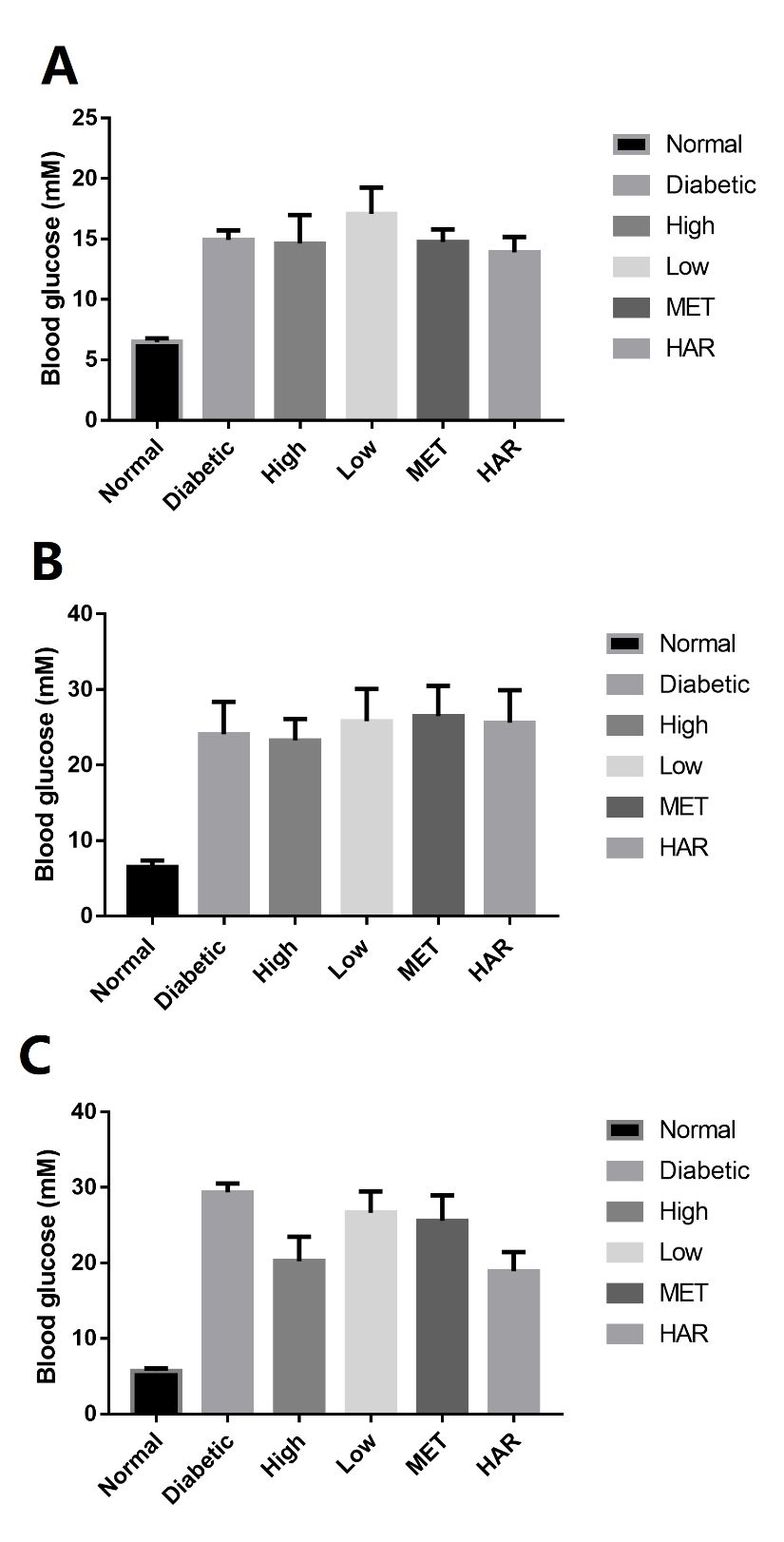


**Figure S8 Random blood glucose levels of *db/db* mice during the experiment.**


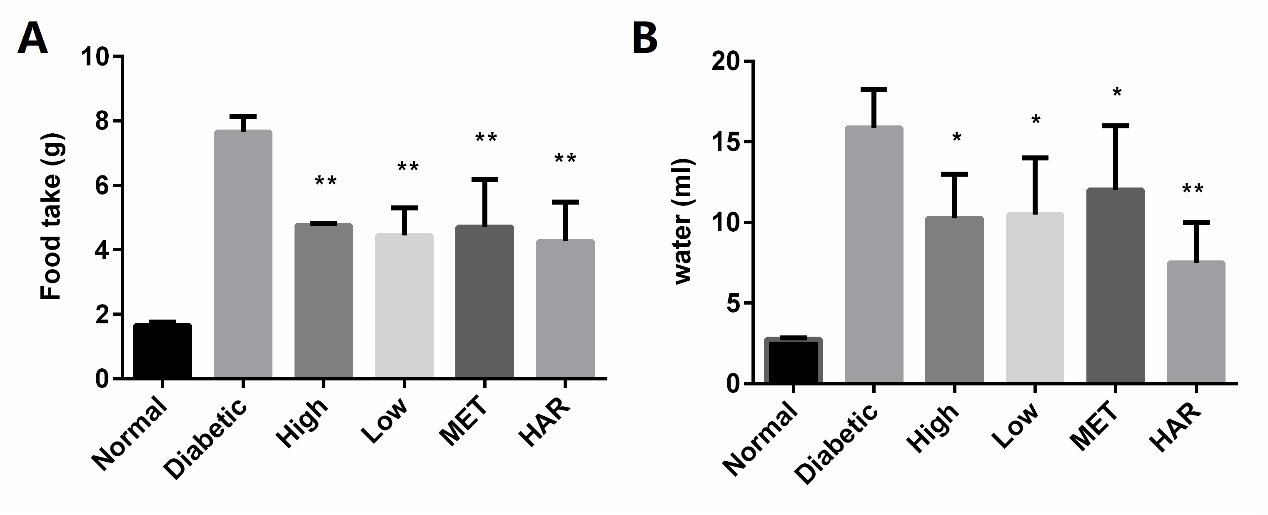


**Figure S9 The levels of (A) Food-intake and (B) Water-intake in each administration group.** Each group had 8 mice, n=8. Data are presented as mean ± SD. Statistical analysis was performed using GraphPad Prism 5.0 with one-way analysis of variance (ANOVA). Differences were statistically significant at *p < 0.05, **p < 0.01. Compared with normal mice, food and water intake in the diabetic model group was significantly increased, which was consistent with the phenomenon of more diet and more water intake in the diabetic model group. After administration, the food and water intake of the db/db mice were reduced, indicating that the drug improved the diabetic symptoms of the mice.

**
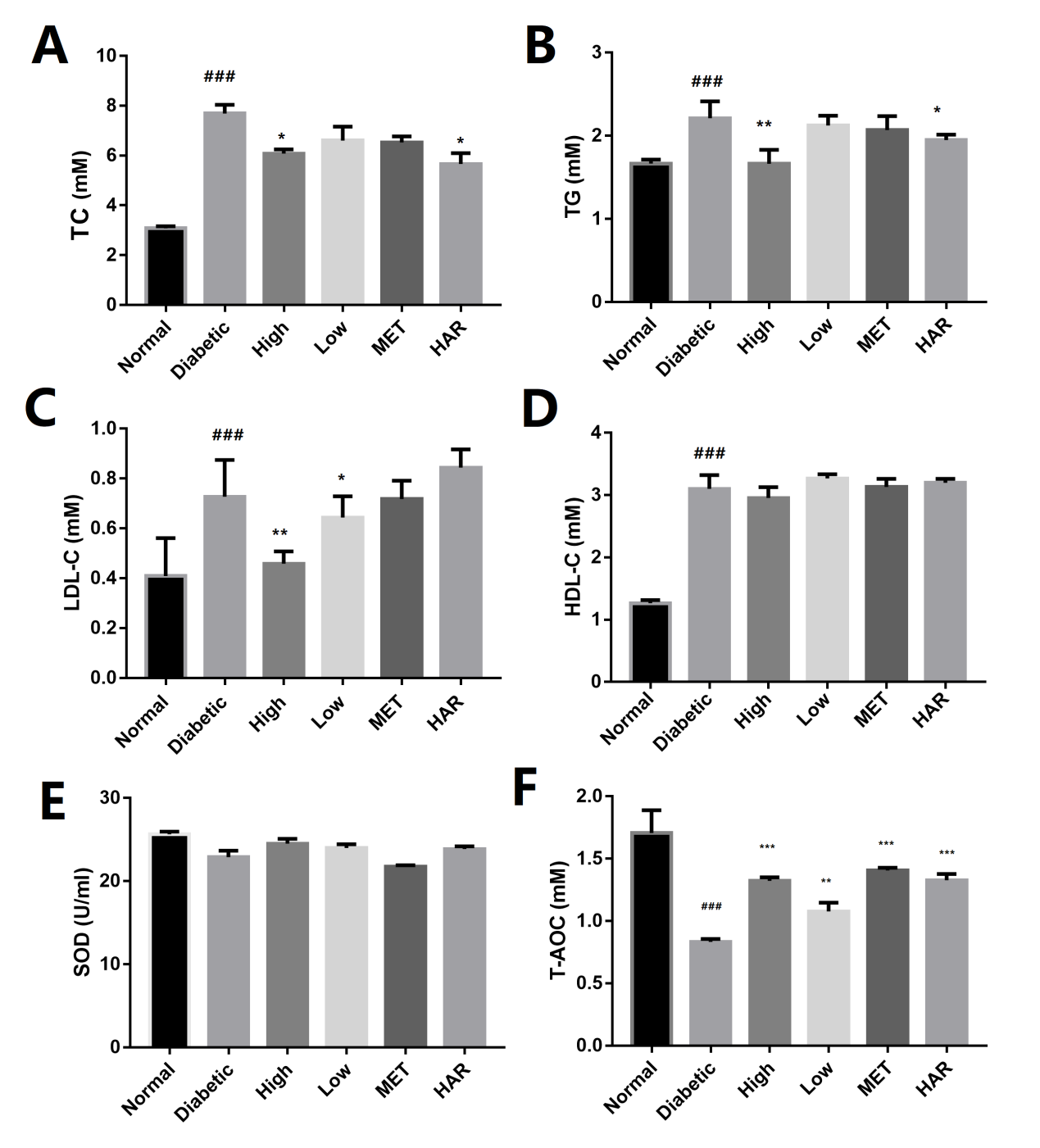
**

**Figure S10 Effects of DMB on serum lipid profiles and oxidative stress regulation in *db/db* mice.** (A-D) The effects of DMB on serum lipid profiles of experimental db/db mice. Dyslipidaemia was ameliorated by DMB treatment in db/db mice. Furthermore, DMB or harmine treatment inhibited the rise in the levels of serum TC, TG, and LDL-C in db/db mice. Moreover, the levels of HDL-C in db/db mice remained unchanged. (E, F) The effects of DMB on the total antioxidant capacity (T-AOC) and serum SOD levels of experiment db/db mice. T-AOC of the high-dose group was increased significantly. Meanwhile, there was no significant difference in serum SOD levels among DMB-treated, metformin-treated, harmine-treated, and untreated diabetic groups. These findings suggest that DMB partially ameliorates anti-oxidative stress in diabetic mice.
