# Supplementary material for: A natural DYRK1A inhibitor as a potential stimulator for β‐cell proliferation in diabetes
Source: Clin Transl Med. 2021 Jul 19;11(7):e494. doi: 10.1002/ctm2.494 (PMC8288015; doi:10.1002/ctm2.494)
Supplement: Supplementary file 13 — Supporting Information 3 [file CTM2-11-e494-s004.docx]

**Supporting Information 3:** Materials and methods

**A natural DYRK1A inhibitor as a potential stimulator for β-cell proliferation in diabetes**

Mengzhu Zheng^1^*, Qingzhe Zhang^1^*, Chengliang Zhang^1,3^*, Canrong Wu^1^, Kaiyin Yang^1^, Zhuorui Song^2^, Qiqi Wang^2^, Chen Li^2^, Yirong Zhou^1^, Jiachun Chen^1†^, Hua Li^1,2†^, Lixia Chen^2†^

**Running title:** DMB can stimulate β-cell proliferation via DYRK1A.

**Affiliations**

^1^ Hubei Key Laboratory of Natural Medicinal Chemistry and Resource Evaluation, School of Pharmacy, Tongji-Rongcheng Center for Biomedicine, Tongji Medical College, Huazhong University of Science and Technology, Wuhan, 430030, China

^2^ Wuya College of Innovation, Key Laboratory of Structure-Based Drug Design & Discovery, Ministry of Education, Shenyang Pharmaceutical University, Shenyang, 110016, China

^3^ Department of Pharmacy, Tongji Hospital, Tongji Medical College, Huazhong University of Science and Technology, Wuhan, 430030, China

* These authors contributed equally to this work.

† Correspondence and requests for materials should be addressed to Lixia Chen (email: [syzyclx@163.com](mailto:syzyclx@163.com)), Hua Li (email: [li_hua@hust.edu.cn](mailto:li_hua@hust.edu.cn)), Jiachun Chen ([homespringchen@126.com](mailto:homespringchen@126.com)).

Hubei Key Laboratory of Natural Medicinal Chemistry and Resource Evaluation, School of Pharmacy, Tongji-Rongcheng Center for Biomedicine, Tongji Medical College, Huazhong University of Science and Technology, Wuhan 430030, P. R. China

Tel: +86-27-83692762 Fax: +86-27-83692762

**Materials and Methods**

***Materials***

Compounds were separated from the plants of *Swertia* genus by our own laboratory and their chemical structures were determined by spectroscopic methods. Specifically, DMB was isolated from the *Swertia bimaculata* (Sieb. et Zucc.) Hook. extract and its chemical structure was identified by comparing its spectroscopic data with those reported in the literature (1). The detailed separation and purification procedure is given as following. The air-dried leaves and stems of *Swertia bimaculata* (40.0 kg) were extracted with 75% EtOH (3 × 40 L × 3 h) to give a total extract after removing solvent *in vacuo*. The extract (1.57 kg) was suspended in water (5 L) and extracted with EtOAc (3 ×5 L). The EtOAc extract (537.0 g) was subjected to silica gel CC eluted with CH_2_Cl_2_-MeOH (100:0, 100:1, 80:1, 70:1, 60:1, 50:1, 40:1, 30:1, 20:1, 3:1, 1:1 and 0:1) to afford eight fractions (F1−F8). Fraction F1 (50.2 g) was subjected to a silica gel CC using cyclohexane−acetone (60:1, 30:1, 20:1, 15:1, 8:1, 5:1) as eluent to yield six subfractions (F11-F16). Subfraction F11 (23.5 g) was crystallized with MeOH to afford compound **1** (DMB, 6.8 g).

Harmine was purchased from Sichuan Weikeqi Biological Technology Co., Ltd. (Sichuan, China). Metformin was purchased from Beijing Jingfeng Pharmaceutical Co., Ltd. (Beijing, China). INS-1 cells were purchased from Shanghai Zhong Qiao Xin Zhou Biotechnology Co., Ltd. (Shanghai, China). The cell lines were authenticated by STR analysis.

Reagents are as follows:

EdU (Guangzhou RiboBio Co., Ltd.); DAPI (Molecular Probes); DYRK1A (D30C10) Rabbit mAb antibody (Cell Signaling Technology, 8765S); NFATc1 antibody (Santa Cruz, CA), anti-insulin antibody (Abcam); anti-glucagon antibody (Abcam); Ki-67 antibody (Cell Signaling Technology); TGFB2 Polyclonal Antibody (Bio-Swamp, PAB33671); Phospho-SMAD3-T179 pAb (Bio-Swamp, PAB43521-P); SMAD3 Polyclonal Antibody (Bio-Swamp, PAB30705); CDKN2A Polyclonal Antibody (Bio-Swamp, PAB36515); E2F1 Polyclonal Antibody (Bio-Swamp, PAB44204); MRPL28 Polyclonal Antibody (Bio-Swamp, PAB34309); CCNA2 Polyclonal Antibody (Bio-Swamp, PAB33497); CORO1A Polyclonal Antibody (Bio-Swamp, PAB36111); CDK1 Polyclonal Antibody (Bio-Swamp, PAB30052); Phospho-H2AFX-S139 pAb (Bio-Swamp, PAB36314-P); FOXO1 Rabbit pAb (abclonal, A13862); PDX1 Rabbit pAb (abclonal, A10173); p-Histone H3 (S10) (Cell Signaling Technology, 9701S); ERK 1 (Santa Cruz , CA, sc-271269); Phospho-p38 MAPK-T180/Y182 Rabbit pAb (AP0526). Chemical reagents were purchased from Sinopharm Chemical Reagent Beijing Co., Ltd (Beijing, China). CCK-8 assay kit was purchased from “Dojindo” (Japan). Insulin ELISA kit was purchased from Bio-Swamp Life Science Lab (Shanghai, Wuhan). Other biochemical kits were purchased from Nanjing Jiancheng Bioengineering Institute (Nanjing, China).

***Molecular docking and structure-based virtual ligand screening***

An in-house small-molecular database containing 158 xanthone compounds was set up by the OpenBabel software (2). The X-ray co-crystal structure of DYRK1A with ligand (PDB code: 4YLK) was chosen as the molecular model for screening (3). Compounds were screened by using ICM-Pro 3.8.1 molecular docking software on an Intel i7 4960 processor (MolSoft LLC, San Diego, CA) (4).

***Protein expression and purification***

The protein expression was performed as described previously with minor modifications (5). Briefly, the truncated DYRK1A residues 127-485 (ACCESSION: NP_001387) was cloned into the pET26b vector (Novagen), and then the recombinant plasmid was transformed into *Escerichia Coli* strain BL21 (DE3) (Invitrogen) after sequence confirmation and selected on kanamycin plates. The transformed cells were grown in Luria-Bertani (LB) media in the presence of kanamycin until the optical density (OD) reached 0.8 at 37 °C. The recombinant DYRK1A protein was over-expressed induced by 0.4 mM IPTG (Isopropyl-β-D-Thiogalactopyranoside) at 16 °C for 16 h. Cells were harvested, and then lysed by ultrasonification on ice in a buffer containing 20 mM Hepes (pH 7.5), 500 mM NaCl, and 5 mM β-mercaptoethanol. Soluble C-terminally hexa-histidine tagged DYRK1A was purified by using a Ni^2+^-chelating column (Qiagen), which was followed by a size exclusion chromatography on a Superdex 300GL column with a buffer containing 20 mM Hepes (pH 7.5) and 500 mM NaCl. The DYRK1A protein was finally concentrated to the appropriate concentration by ultra filtration (Millipore).

***Crystallization and structure determination***

Crystals of the DYRK1A complex with DMB were obtained by co-crystallization with sitting drop vapor diffusion method. Purified DYRK1A was concentrated to 15 mg/mL, then incubated with DMB at molar ratio of 1:3 over ice for 1 h. 1 μL of DYRK1A-DMB solution was mixed with 1 μL of mother liquor, further equilibrated with reservoir solution at 4 °C. Crystals appeared in a week, with crystallization condition of 0.1 M Bis-Tris, pH 6.0, 17% PEG1500, and subsequently soaked in the crystallization solution with 1 mM DMB again. This was followed by cryoprotection using the crystallization solution with the addition of 30% ethylene glycol, followed by flash freezing directly into liquid nitrogen.

Data were collected at the Shanghai Synchrotron Radiation Facility (SSRF), and processed by HKL2000. The structure of DYRK1A-DMB complex was solved by molecular replacement (MR) using the program PHASER in the PHENIX package (6) with the search model of PDB 4YLK, followed by repeated cycles of model building with Coot (7) and refinement with REFMAC (8) and PHENIX, yielding the published DYRK1A-DMB complex structure (PDB ID 6LN1).

***In vitro enzyme inhibition assay***

The inhibition activity on DYRK1A was assayed by monitoring the decrease of NADH in ultraviolet absorption (λ_max_ = 340 nm) by Synergy Multi-Mode Microplate Reader (BioTek, USA). The substrate peptide (RARPGTPALRE) was synthesized from Wuhan Bioyeargene Biotechnology Co., Ltd (Wuhan, China). The reaction mixture consisted of 0.25 mM substrate peptide, 25 U/mL LDH, 10 U/mL pyruvate kinase, 1 mM phosphoenolpyruvate, 0.2 mM NADH, 25 mM Hepes (pH 7.5), 150 mM NaCl, 5 mM MgCl_2_, 1 mM ATP and 0.4 μΜ DYRK1A. For inhibition experiment, compounds were co-incubated with the protein for 15 min. Next, the reaction was initiated when adding the above solutions. The decrease rate of NADH consumed in the reactions was monitored kinetically under 30 °C for 10 min using a BioTek Synergy HT microplate reader. The IC_50_ values of candidates were calculated by using a standard dose response curve fitting by importing these data into Prism (version 6.02, GraphPad)

***Microscale thermophoresis (MST) assay***

The MST assay was performed according to the supplied labeling protocol. The recombinant DYRK1A protein was labeled with Monolith NT^TM^ Protein Labeling Kit RED (Cat#L001). After protein purification, the labeled DYRK1A concentration was adjusted to 200 nM. Then, a series of dilution solution of DMB (**1**) and harmine (5-10000 µM) in the same buffer (20 mM Hepes, pH 7.5) were prepared. The solution was mixed with the labeled DYRK1A protein in the ratio of 1:1. After incubation for 15 minutes at room temperature, all samples were loaded into Monolith^TM^ standard glass capillaries and immediately measured by MST with a LED power of 100%, a MST power of 40 % at 25 °C. The dissociation constant K_d_ values were calculated with curve fitting by using NT Analysis software (NanoTemper Technologies, München, Germany).

***Cell culture***

INS-1 cells were cultured in RPMI 1640 containing β-mercaptoethanol (50 μM), 10 % fetal calf serum, penicillin (100 U/mL), streptomycin (0.1 mg/mL) in a saturated humidified atmosphere of 5% CO_2_ at 37 °C. The culture medium was refreshed every 24 h.

***Cellular thermal shift assay***

Briefly, INS-1 cells cultured in 6-well plates at 90% confluence were treated with DMSO or DMB for 6 h. Then cells were harvested and washed with 0.9% NaCl. The cell suspensions were heated for 5 min to 44, 46, 48, 50, 52, 54 °C, lysed using liquid nitrogen, and freeze-thawed for three times. Soluble proteins were separated from the precipitated fraction by centrifugation at 17,000 g for 20 min and were kept at −80 °C until Western blot analysis.

***RNA interference***

For siRNA transfections, INS-1 cells were transfected with 60 nM siRNA duplex (DYRK1A^#^1 and DYRK1A^#^2) and Lipofectamine 2000 following the manufacturer's instructions (Invitrogen). Then, cells were cultured for 72 h after different treatment. Single siRNA duplexes, synthesized by GenePharma (Shanghai), were:

5'-UAAGGAUGCUUGAUUAUGATT-3' (DYRK1A ^#^1),

5'-AAACUCGAAUUCAACCUUATT-3' (DYRK1A ^#^2), and

5'-UUCUUCGAACGUGUCACGUTT-3' (NC)

***Cell proliferative assay***

INS-1 cells (2 x 10^6^/well) were seeded into confocal dishes (NEST, Jiangsu, China). After incubation at 37 °C for 12 h, the culture medium was changed to medicated culture medium containing EdU (25 μM) and DMB at the concentrations of 0 μM, 37.5 μM, and 75 μM, respectively. The cell was incubated for another 48 h. In the streptozotocin (STZ) induced β-cell damage model, the cells were exposed to 20 mM streptozotocin for 2 h after seeded into confocal dishes. Then, the culture medium was changed to medicated culture medium containning EdU (25 μM) and DMB at the concentrations of 0 μM, 37.5 μM, and 75 μM, respectively. The cell was incubated for another 48 h. The medium was refreshed every 24 hours.

***EdU incorporation and Click-iT^TM^ reaction***

The EdU incorporation experiment was performed according to the instruction of the Click-iT^TM^ EdU Apollo Stain Kit (Invitrogen, OR, USA) with minor modifications. In brief, the harvested cells were washed with PBS and then fixed with 200 μL 4 % paraformaldehyde for 30 min. Cells were washed again and incubated with 200 μL permeabilization buffer containing 0.5 % Triton X-100 for 10 min. After additional washing, cells were incubated with 150 μL Click-iT reaction buffer in dark for 1 h and washed again with permeabilization buffer and PBS, respectively. For subsequent DNA staining, cells were incubated with Hoechst 33342 (5 μg/mL) for another 30 min. The confocal dishes were visualized by laser confocal microscopy (OLYMPUS, Japan). All steps were carried out at 20 °C.

***RNA extraction and qRT-PCR***

INS-1 cell RNA was extracted using Trizol Reagent (Invitrogen). Total RNA was prepared according to the standard RNeasy protocol. cDNA reverse transcription was performed using PrimeScriptTMRT reagent Kit with gDNA Eraser. And Real-time PCR was performed using SYBR® premix Ex Taq^TM^ kit (TaKaRa) on the StepOne^TM^ Real-Time PzCR system. Cycling conditions were 95 ˚C for 1 min, followed by 40 cycles of 95 ˚C for 15 s, 58 ˚C for 20 s, and 72 ˚C for 45 s. Primers used were β-actin: CGTTGACATCCGTAAAGACCTC and TAGGAGCCAGGGCAGTAATCT; Ccnd1: AGTTCATTTCCAACCCACCCT and GAAAGTGCGTTGTGCGGTAG; Ccnd2: GAGTCCCGACTCCTAAGACCC and TTGCGAAGGATGTGCTCAAT; Ccnd3: GGCTATGAACTACCTGGATCGC and AGAGGCGGTGCAGAATCAAG; CDK4: GGCTTGCCTGTTGAGACCAT and GCAGAAGAACTTCAGGAGCCC.

***Microarray analysis of the mRNA expression***

(1) RNA extraction and purification

Total RNA was extracted using TRIZOL Reagent (Cat#15596-018, Life technologies, Carlsbad, CA, US) following the manufacturer’s instructions and checked for a RIN number to inspect RNA integrity by an Agilent Bioanalyzer 2100 (Agilent technologies, Santa Clara, CA, US). Qualified total RNA was further purified by NucleoSpin RNA Clean-up XS kit (Cat#740903, MN, Germany) and RNase-Free DNase Set (Cat#79254, QIAGEN, GmBH, Germany).

(2) RNA amplification and labeling

Total RNA were amplified, labeled and purified by using GeneChip® 3' IVT PLUS Reagent Kit (Cat#902416, Affymetrix, Santa Clara, CA, US) followed the manufacturer’s instructions to obtain biotin labeled cRNA.

(3) Array hybridization

Array hybridization and wash was performed using GeneChip® Hybridization, Wash and Stain Kit (Cat#900720, Affymetrix, Santa Clara, CA, US) in Hybridization Oven 645 (Cat#00-0331-220V, Affymetrix, Santa Clara, CA, US) and Fluidics Station 450 (Cat#00-0079, Affymetrix, Santa Clara, CA, US) followed the manufacturer’s instructions.

(4) Data acquisition

Slides were scanned by GeneChip® Scanner 3000 (Cat#00-00212, Affymetrix, Santa Clara, CA, US) and Command Console Software 4.0 (Affymetrix, Santa Clara, CA, US) with default settings. Raw data were normalized by MAS 5.0 algorithm, Affy packages in R.

For gene expression assays, cDNA was synthesized with random hexamers using a high-capacity cDNA reverse transcription kit (Life Technologies), followed by real-time PCR quantification using TaqMan primers.

***Animal experiments***

All experiments were conducted in compliance with the Guide for the Care and Use of Laboratory Animals of Huazhong University of Science and Technology (Wuhan, China) and approved by the Ethics Committee. Six week-old male db/db mice (B6.BKS (D)-Leprdb/Nju) and lean wild type littermates were purchased from the Model Animal Research Center of Nanjing University. Animals were housed in the animal care facility of Tongji Medical college under standard conditions at 25 °C, humidity of 60 ± 5 % under 12 hours dark/light cycle. Mice had free access to water and food throughout the study period. After 7 days acclimation, diabetic mice were randomly divided into six groups (n = 8). In the experiment, a total of 48 mice (8 normal mice, 40 diabetic mice) were used. Group 1: the control group, normal mice were orally administrated with distilled water (Vehicle); Group 2: the vehicle group, diabetic mice were orally administrated with distilled water (Vehicle); Group 3: the metformin-treated group, diabetic mice were orally administrated with positive drug metformin (200 mg/kg BW/D); Group 4: the harmine-treated group, diabetic mice were orally administrated with harmine (200 mg/kg BW/D); Group 5: the DMB low-dose group, diabetic mice were orally administrated with DMB (75 mg/kg BW/D); Group 6: the DMB high-dose group, diabetic mice were orally administrated with DMB (150 mg/kg BW/D). The experimental period was six weeks. Three mice were selected in each group, which were injected with EdU intraperitoneally at a dose of 5 mg/kg/BW for 10 days.

***The measurements of body weight, food intake and blood glucose level***

Body weight, food-intake and blood glucose level of mice were monitored weekly. Glucose measurements were performed on blood drawn from the tail vein using a Bayer Contour Glucose Meter (Bayer, Germany).

***Oral glucose tolerance test (OGTT)***

OGTT was measured at 38 days (the sixth week). When the db/db mice and normal mice were fasted for 6 h (but not water), they were intragastrically administrated with glucose (2.5 g/kg/BW). Blood samples were collected from the tail vein at 0, 0.5, 1, 1.5, 2 h after glucose loading, and the blood glucose levels of all samples were immediately measured by using a Bayer Contour Glucose Meter.

***Fasting serum insulin levels and antioxidant parameter levels in mice***

The mice were sacrificed after treatment for 6 weeks. Blood samples and tissues for bioassay were obtained from 12 h fasting mice. Blood samples were centrifuged (4000 rpm, 15 min) and then stored at -20 °C for further study. Fasting serum insulin level was measured by the Mouse insulin ELISA kit (TSZ, USA). According to the kit instructions, the activity of serum superoxide dismutase (SOD) and total antioxidant capacity (T-AOC) were respectively analyzed, along with the level of serum glutathione (GSH). All assay kits were purchased from Jiancheng Bioengineering Institute (Nanjing, China).

***Western blot analysis***

Proteins (40 μg/sample) were lysed in RIPA lysis buffer. Insoluble debris was removed by centrifugation at 12,000 rpm for 5 min, and the content of protein was determined using Bradford reagent (Bio-Rad, USA). Then the lysate protein was subjected to 12 % SDS-PAGE and transferred to polyvinylidene difluoride membranes (PVDF) (Millipore). After blocked with 5 % non-fat milk for 1 h, the membranes then incubated with the respective specific primary antibody at 4 °C overnight. After incubated with secondary antibody, protein bands were visualized using an enhanced chemiluminescence reagent (ECL Plus) (GE Healthcare, USA). And the AlphaEaseFC software was used to analyze the optical density of the target strip.

***Histochemical examination of EdU labeling pancreas tissues***

After 32 days of administration, the mice were injected with EdU solution (dissolved in normal saline) at a dose of 5 mg/kg, and each group was given three mice for 10 days. The mouse pancreas tissues were removed after the mice were sacrificed. All tissue samples were divided into two parts, one of which was stored at -80 °C for further study, and the other one were stored in 4 % paraformaldehyde after washing with PBS buffer. The tissues were embedded in paraffin, sectioned, and then stained by using double immunofluorescence method for histopathological assessment through microscopic observation (Olympus, Tokyo, Japan) or by using the Click-iTTM EdU Apollo Stain Kit.

***Statistical analysis***

Data are presented as mean ± SD. Statistical analysis was performed using GraphPad Prism 5.0 with one-way analysis of variance (ANOVA). Differences were statistically significant at **p* < 0.05, ***p* < 0.01.

**References**

1. Liu Z, Wan L, Yue Y, et al. Hypoglycemic Activity and Antioxidative Stress of Extracts and Corymbiferin from Swertia bimaculata In Vitro and In Vivo. *Evid Based Complement Alternat Med*. 2013;2013:125416.

2. O'Boyle NM, Banck M, James CA, et al. Open Babel: An open chemical toolbox. *J Cheminform*. 2011;3:33.

3. Falke H, Chaikuad A, Becker A, et al. 10-iodo-11H-indolo[3,2-c]quinoline-6-carboxylic acids are selective inhibitors of DYRK1A. *J Med Chem*. 2015;58(7):3131-3143.

4. R. Abagyan MTDK. ICM-A New Method for Protein Modeling and Design: Applications to Docking and Structure Prediction from the Distorted Native Conformation. *J Comput Chem* 1994; 5: 488-506

5. McCoy AJ, Grosse-Kunstleve RW, Storoni LC, et al. Likelihood-enhanced fast translation functions. *Acta Crystallogr D Biol Crystallogr*. 2005;61(Pt 4):458-464.

6. Adayev T, Wegiel J, Hwang YW. Harmine is an ATP-competitive inhibitor for dual-specificity tyrosine phosphorylation-regulated kinase 1A (DYRK1A). *Arch Biochem Biophys*. 2011;507(2):212-218.

7. Emsley P, Cowtan K. Coot: model-building tools for molecular graphics. *Acta Crystallogr D Biol Crystallogr*. 2004;60(Pt 12 Pt 1):2126-2132.

8. Murshudov GN, Vagin AA, Dodson EJ. Refinement of macromolecular structures by the maximum-likelihood method. *Acta Crystallogr D Biol Crystallogr*. 1997;53(Pt3):240-255.
